# Modulation of Respiration and Mitochondrial Dynamics by SMAC-Mimetics for Combination Therapy in Chemoresistant Cancer

**DOI:** 10.7150/thno.33758

**Published:** 2019-07-09

**Authors:** Judith Hagenbuchner, Herbert Oberacher, Kathrin Arnhard, Ursula Kiechl-Kohlendorfer, Michael J. Ausserlechner

**Affiliations:** 1 Department of Pediatrics II, Medical University Innsbruck, Innsbruck, Austria; 2 Department of Pediatrics I, Medical University Innsbruck, Innsbruck, Austria; 3 Institute of Legal Medicine and Core Facility Metabolomics, Medical University Innsbruck, Innsbruck, Austria

**Keywords:** BIRC4/XIAP, BIRC5/survivin, Warburg, SMAC-mimetics

## Abstract

Inhibitor of apoptosis proteins (IAP) are cell death regulators that bind caspases and interfere with apoptotic signalling *via* death receptors or intrinsic cell death pathways. BIRC4/XIAP is the most potent anti-apoptotic IAP-member and it physically interacts with caspases *via* its BIR2 and its BIR3 domain. These domains are also critical for the interaction with mitochondria-derived SMAC/Diablo and with the IAP protein survivin. Survivin is frequently overexpressed in neuroblastoma due to a gain of 17q and we have demonstrated that survivin confers resistance to chemotherapeutic agents and reprograms metabolism of neuroblastoma cells towards glycolysis. As regulator of mitochondrial fission and autophagy survivin acts at the crossroads of mitochondrial architecture, autophagy and cellular energy metabolism.

**Methods**: We tested the effect of SMAC-mimetics on the XIAP/survivin axis as modulator of cellular metabolism analysing mitochondrial morphology, metabolic intermediates and cellular survival. Finally, the impact of the combined treatment was evaluated in a xenograft neuroblastoma mouse model assessing the therapy effect on tumour size and volume.

**Results**: Here we demonstrated that XIAP sequesters significant amounts of survivin within the cell that can be mobilized by so called SMAC-mimetics. SMAC-mimetics are drugs that are designed to bind with high affinity to XIAP-BIR2 / BIR3 domains to release caspases and re-sensitize XIAP-overexpressing tumors for chemotherapy. However, SMAC-mimetic treatment releases also survivin from XIAP and thereby induces mitochondrial fragmentation, prevents ROS accumulation and leads to the Warburg effect, an unwanted side effect of this therapy. Importantly, cells that drift into a highly glycolytic state due to SMAC-mimetic treatment become also highly sensitive to non-genotoxic treatment with glycolysis inhibitors such as 2-Deoxy-D-glucose (2DG) *in vitro* and *in vivo*.

**Conclusion**: A combinational therapy of non-genotoxic SMAC-mimetics and glycolysis-inhibitors overcomes IAP-mediated cell survival in cancer and provides therefore an attractive usage of SMAC-mimetics.

## Introduction

Inhibitor of apoptosis proteins (IAPs) act as key regulators in apoptosis by directly or indirectly affecting cell death signaling. BIRC5/survivin is the smallest IAP member and its main functions are the regulation of cell division, the inhibition of cell death and its involvement in DNA-double-strand repair 1, 2. Survivin is highly expressed in many different tumor types and its elevated expression correlates with enhanced chemotherapy resistance. This is especially true for high-stage, aggressive, neuroblastoma where overexpression of survivin is frequently caused by a gain of chromosome 17q, suggesting that this protein represents a promising therapeutic target in malignant cells derived from neuronal precursors 3, 4. However, several attempts to neutralize or lower survivin by systemic administration of drugs have demonstrated severe side effects in patients 5-8 or failed due to lack of specificity 9. Therefore, systemic targeting and neutralization of survivin might not be the strategy of choice, especially not for the treatment of young pediatric patients. Interestingly, survivin interacts with the cancer-related IAP BIRC4/XIAP, which increases the half-life of both proteins and thereby may contribute to the anti-apoptotic function of survivin 10 and XIAP. However, in presence of XIAP-associated factor 1 (XAF1), which activates the XIAP E3-ubiquitin ligase activity, the survivin-XIAP interaction may also lead to the degradation of survivin. Therefore, depending on cell type-specific co-factors, XIAP can contribute to either survivin stabilization or to its proteasomal degradation 11. XIAP is overexpressed in a variety of human cancers and mediates resistance to chemotherapeutic drugs in specific subgroups of patients (reviewed in 12). To overcome XIAP-mediated cell death inhibition, several small molecules derived from the N-terminal peptide sequence of the XIAP-interacting protein Second mitochondria-derived activator of caspases (SMAC/Diablo) have been developed. The N-terminal peptide sequence of SMAC binds to the BIR3-domain of XIAP with a KD of 244 nM 13, whereas the interaction between SMAC and Survivin-BIR domain is 500fold lower at a KD of 121 µM 14, suggesting that SMAC-derived peptidomimetics (SMAC-mimetics) optimized for XIAP-BIR3-binding at KDs in the low nM range show no high affinity to survivin. SMAC-mimetic binding to XIAP releases sequestered caspase 9 (CASP9) and thereby should facilitate cell death. However, due to the similarity of XIAP-, cIAP1-and cIAP2-BIR3 domains, also cIAP1/2 are efficiently targeted, which induces their proteasomal degradation and TNF-mediated cell death in cancer cells 15, 16. Several of these compounds are currently tested in clinical trials 17. In childhood neuroblastoma, SMAC-mimetics significantly affect tumor growth and death resistance of cancer cells *in vitro* and *in vivo* 18, 19. Although SMAC-mimetics have been evaluated for several years now, their safe application in clinic is still under debate and a reliable biomarker for patient selection and therapy monitoring is still missing (reviewed in 20). Survivin, the smallest member of the IAP family contains a BIR domain, but lacks all other domains found in IAP proteins, has an indispensable function in cell division and in cell death regulation (reviewed in 2). It is well established that survivin, Aurora B, Borealin and INCENP form the so called chromosomal passenger complex that controls spindle assembly and chromosomal segregation during cell division (reviewed in 21). On the other hand, Survivin via XIAP interaction may promote metastasis 22 and protect against cell death 4, 23. In recent publications, we demonstrated that survivin is a critical regulator of chemo-resistance and cellular metabolism that changes mitochondrial structure and activity and shifts neuroblastoma cells into a chemo-resistant, highly glycolytic state 4, 24. This in turn renders these cells highly sensitive to treatment with glycolysis-inhibitors *in vitro* and *in vivo* 24, 25. We now discovered that XIAP sequesters significant amounts of cellular survivin in neuroblastoma cells. Neutralization of XIAP for chemotherapy-sensitization by SMAC-mimetics displaces survivin and causes the above phenomenon of a survivin-induced “Warburg effect” - i.e. increase of glycolytic activity and the shutdown of ROS-producing respiratory complexes. Our results therefore uncovered a dark-side of SMAC-mimetics, i.e they increase the resistance to DNA-damaging chemotherapeutic agents upstream of XIAP rather than sensitizing for them. By co-administration of glycolysis-inhibitors which antagonize survivin expression, we were able to overcome IAP-mediated cell survival and to induce cell death in a variety of cancer cells. Therefore, the combination of these two non-genotoxic substances offers an interesting therapeutic window in the treatment of malignancies with deregulated IAPs.

## Materials and Methods

### Cell lines, culture conditions, and reagents

The neuroblastoma lines SH-EP, STA-NB15, LAN-1, as well as the colon carcinoma cell line LoVo, the leukemia cell line CEM-C7H2 and Phoenix^TM^ packaging cells 26 were cultured in RPMI1640 (Lonza, Basel, Switzerland). The neuroblastoma cell line NxS2 and the cell line HEK293T were cultured in DMEM (Invitrogen, Carlsbad, CA, USA). The neuroblastoma cell line SH-SY5Y was cultured in DMEM/F12 (Invitrogen, Carlsbad, CA, USA). All media contain 10% fetal bovine serum (Sigma- Aldrich, Vienna, Austri), 100 U/ml penicillin, 100 µg/ml streptomycin and 2 mM L-glutamine (Lonza, Basel, Switzerland). All cultures were routinely tested for mycoplasma contamination using the Venor^R^ GeM-mycoplasma detection kit (Minerva Biolabs, Berlin, Germany). All reagents were purchased from Sigma-Aldrich (Vienna, Austria) unless indicated otherwise. The SMAC-mimetics were purchased from Active Biochem (Wan Chai, HongKong, People's Republic of China).

### Expression vectors and genetically modified cell lines

The retroviral vectors coding for pLIB-Survivin- iresYFP, and the lentiviral vectors pLKO.1-Puro, pLKO-shSurvivin-Puro, and pLKO-shDrp1-Puro have been described previously 4, 27. The vectors pLKO-shXIAP-Puro (TRCN0000003785-8, and TRCN0000010817) were purchased from Sigma-Aldrich (Vienna, Austria), SH-EP/Ctr, and SH-EP/Surv cells have been described previously 24. SH-EP/Surv cells were sorted by FACS for YFP-positive cells. pLKO.1 served as control (SH-EP/shCtr) for knock- down cells for Survivin (SH-EP/shSurv), DNML1/ Drp1 (SH-EP/shDrp1) 24 and XIAP (SH-EP/ shXIAP). SH-EP/shXIAP cells were generated by bulk infection with pLKO-shXIAP vectors and after Puromycin selection raised as single cell clones as described in Hagenbuchner et al^25^.

### Subcellular fractionation, co-immunoprecipitation and immunoblotting

For immunoblot analyses total protein or cytoplasmic and mitochondrial extracts were prepared as described previously 25, 28. Co-immunoprecipitation was performed as described in 25, 29, 30 using either 1 µg anti-Survivin antibody, anti-XIAP antibody or normal IgG as control covalently coupled to Affi-Prep Protein A support (Bio-Rad, Munich, Germany). Total protein (50 µg/lane), cytoplasmic and mitochondrial fractions (20 µg/lane) or immunoprecipitates as well as input controls were separated by SDS-PAGE and blotted. Membranes were blocked, incubated with primary antibodies against α-Tubulin, BCL2L11/Bim, cIAP1, cIAP2, XIAP (Cell Signaling Technology Inc., Boston, USA), Survivin (Upstate Biotechnology, Lake Placid, USA), OXPHOS, and CoxIV, DRP1, and pDRP1-Ser637 (Abcam, Cambridge, UK), washed and detected with secondary horseradish-peroxidase-conjugated antibodies. The blots were developed by enhanced chemiluminescence (GE-Healthcare, Vienna, Austria) and measured with an AutoChemi detection system equipped with LabWorks software (UVP, Cambridge, UK).

### Live cell fluorescence microscopy

For live cell analyses cells were grown on glass slides or LabTek Chamber Slides^TM^ (Nalge Nunc International, USA) coated with 0.1 mg/ml collagen. Mitochondrial morphology was analyzed by CMXros- staining (300 nM; Thermo Fisher Scientific, Waltham, USA). Mitochondrial ROS generation was visualized by reduced MitoTrackerRed/CM-H_2_XRos staining (500 nM, Thermo Fisher Scientiftic, Waltham, USA). Images were collected using an Axiovert200M microscope equipped with an ApoTome.2 system (Zeiss, Vienna, Austria). Fluorescence intensity was quantified using Axiovision Software from at least 30 cells (Zeiss, Vienna, Austria) and relative ROS levels were expressed as % of untreated controls.

### Metabolic assays

Viability of cells was determined by resazurin reduction assay in a Chameleon MicroplateReader (Hidex, Turku, Finnland) 31. Extracellular Glucose- and Lactate-amounts were measured after cultivation of identical cell numbers for 72 hours by colorimetric method using the Glucose or Lactate Assay Kit (BioVision, Mountain View, USA) according to the manufacturer's instruction. All levels were normalized to cell numbers.

### Intracellular metabolites

Cells were grown at identical density with or without 5 µM LCL161. 8x10^7^ cells per ml were resuspended in phosphate buffer /EtOH buffer (15/85%; v/v%). Lysis was performed by three rounds of sonification (Sonopuls, Bandelin electronics, Berlin, Germany), N2 freezing, and heating to 98 °C. The supernatant was collected for quantitative analysis of intracellular levels of glucose, pyruvic acid, citric acid, succinic acid, fumaric acid and malic acid. Quantitation was accomplished by gas chromatography-mass spectrometry (GC-MS) using methanolic standard solutions of the targeted compounds and nonadecanoic acid as internal standard for calibration.

Glucose, pyruvic acid, citric acid, succinic acid, fumaric acid, malic acid, nonadecanoic acid, pyridine, methoxyamine hydrochloride, N-methyl-trimethylsilyl-trifluoroacetamide (MSTFA) were obtained from Sigma-Aldrich (St. Louis, USA).

Dried extracts or the dried metabolite standard mixes were derivatized to its (Meox-)TMS-derivatives through 2 h reaction with 30 µL of 20 mg/mL methoxyamine hydrochloride solution in pyridine, followed by a 1 h reaction with 60 µL of MSTFA, both at 60 °C.

The GC-MS system consisted of a HP7890 GC device with a HP5975C inert XL mass-selective detector (Agilent Technologies, Santa Clara, CA, USA). A DB-XLB column (30 m x 0.25 mm i.d. x 0.25 µm film thickness, J&W Scientific, Folsom, CA) was used for chromatographic separation. Carrier gas was helium with a flow rate of 1.0 ml/min. Injection volume was 1 µl (splitless), injection temperature was 250 °C. The temperature program was as follows: 50 °C, hold 2 min; heat to 310 °C with 10 °C/min, hold for 10 min. MS was done in electron impact mode (70 eV) scanning from 50 to 800. Mass spectral data were recorded on a personal computer with the HP MS ChemStation software G1034C version D01.00 (Agilent Technologies). Data mining was accomplished with Quantitative Analysis of MassHunter Workstation Software (Agilent Technologies).

### Cellular respiration

Analyses of respiration and inhibition/ uncoupling of respiratory chain complexes were done using Agilent Seahorse XFp cell culture miniplates and Seahorse XFp Cell mito stress test kit (Agilent Technologies, Santa Clara, CA, USA) in a Seahorse XFp System (Agilent Technologies, Santa Clara, CA, USA) according to manufacturer's instructions.

### Mouse Xenograft

All mice were maintained under specific pathogen-free conditions at the Central Laboratory Animal Facility of the Medical University Innsbruck. The animal experiments were approved by the Austrian Federal Ministry of Science and Research (BMWFW-66.011/0112-WF/V/3b/2014) and carried out in accordance with European standards, the Austrian Law on Animal Experiments and the Austrian Experiments Regulation. 7-weeks old Balb c nu/nu mice were obtained from Envigo (Huntigdon, Great Britain) and were engrafted with 2x10^7^ LAN-1 cells into each flank in 0.1 ml 50% Matrigel (Corning, New York, USA). Seven to nine days later, tumor-bearing mice were randomly divided into four treatment groups: 4 mg/kg LCL161 (i.p), 5 mg/0.2 ml (i.p) 2-Deoxy-D-Glucose, LCL161/2DG combination or PBS/EtOH (v:v = 90:10). Injections were given over a period of three weeks three times per week. Tumor size was measured three times weekly with the help of an electronic caliper (volume=(length * width^2^)/2). Afterwards, the tumor weight was measured. The study used 6 mice per group and statistical comparison of tumor volume and weight used the Mann-Whitney-U-test.

### Statistical analyses

Statistical significance of differences between controls and treated cells were calculated using unpaired t-test. For animal studies the Mann- Whitney-U-test was used. All statistical analyses were performed using Graph Pad Prism 8.0 software.

## Results

**Figure 1:**
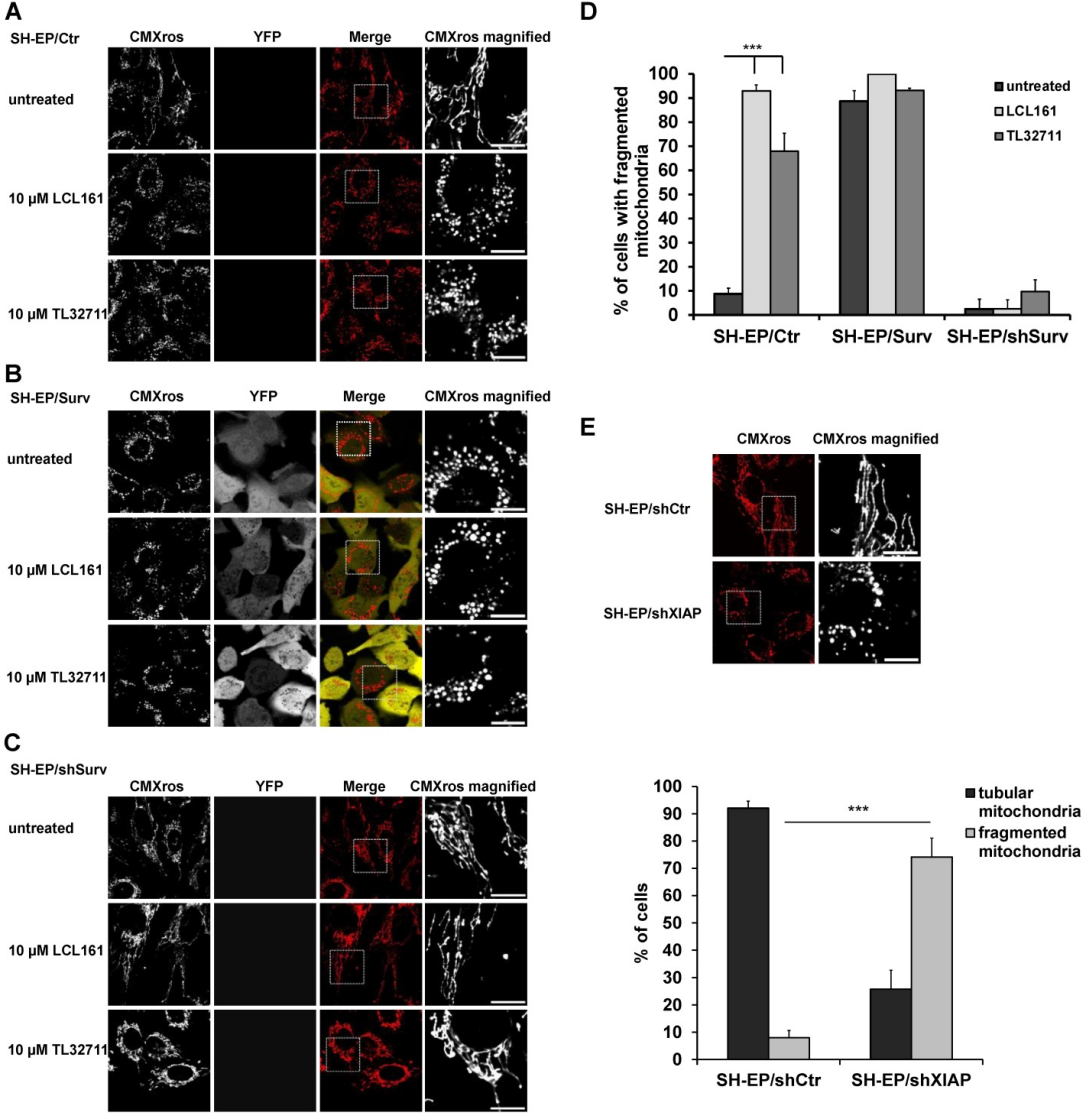
** SMAC-mimetics induce survivin-dependent mitochondrial re-organisation.** SH-EP/Ctr (**A**), SH-EP/Surv (**B**) or SH-EP/shSurv (**C**) cells were seeded on glass slides and treated for 24 hours with either 10 µM LCL161 or 10 µM TL32711. Mitochondria were stained with 300 nM CMXRos and analyzed with a 63x oil objective in an Axiovert200M microscope equipped with an ApoTome.2 system. Shown are representative images. Bar is 10 µm. (**D**) For quantification, at least 60 cells from three independent experiments were analyzed for mitochondrial morphology. ***P<0.001. (**E**) Mitochondrial staining of SH-EP/shCtr and SH-EP/shXIAP cells (CMXRos 300 nM). Bar is 10 µm. For quantification at least 90 cells out of four independent experiments were analyzed for tubular or fragmented mitochondria. ***P<0.001.

We recently discovered that Survivin, when amplified or ectopically expressed, affects mitochondrial morphology by recruiting the fission protein DNML1/DRP1 to mitochondria. Survivin-induced mitochondrial fission does not result in apoptosis but in a metabolic shift to glycolysis and downregulation of the respiratory complexes I, II and IV 24. Since survivin stability is in part regulated by interaction with XIAP, we wondered whether treatment with XIAP-inhibitory substances (SMAC-mimetics) also affects mitochondrial morphology and metabolism. Therefore, we treated SH-EP/Ctr and SH-EP/Surv cells for 24 hours with the already clinically tested SMAC-mimetics LCL161, TL32711/birinapant (bivalent), AT-406, and CUDC-427/GDC-0917 and monitored mitochondrial morphology by live-cell fluorescence microscopy. To ensure that mitochondrial structures were not affected by cell death, we chose a non-toxic concentration, as verified by PI-FACS analyses over a time period of 48 hours (**Supplementary Figure S1A and S1B**). As shown in **Figure 1A, Figure 1D** and **Supplementary Figure S2A**, SMAC-mimetics lead to fragmentation of the mitochondrial networks within 24 hours in SH-EP/ Ctr cells comparable to mitochondrial structures in cells with ectopic survivin expression (**Figure 1B and 1D**). This effect seems so be concentration and time-dependent, since also 100 to 1000 times lower concentrations increase the amount of mitochondrial fragmentation when applied for 72 hours (**Supplementary Figure S1C and S1D**). To further prove that this effect depends on survivin and DRP1 recruitment, we tested the effect of LCL161 and TL32711 on mitochondria in survivin or DRP1 knock-down cells. In these cells SMAC-mimetics failed to induce mitochondrial fragmentation (**Figure 1C, 1D and Supplementary Figure S2B**). Besides protein knock-down also the chemical reduction of survivin by the transcription-inhibitory compound YM155 reverted the effect of LCL161 on mitochondrial structures (**Supplementary Figure S2C**), providing a functional link between survivin expression levels and the effect of SMAC-mimetics on mitochondrial morphology. Also the knock-down of XIAP itself (**Supplementary Figure S2D**) leads to fragmentation of mitochondria (**Figure 1E**) suggesting that disruption of the XIAP/survivin complex is essential for survivin's function at the mitochondria.

### SMAC-mimetics release survivin from XIAP

**Figure 2:**
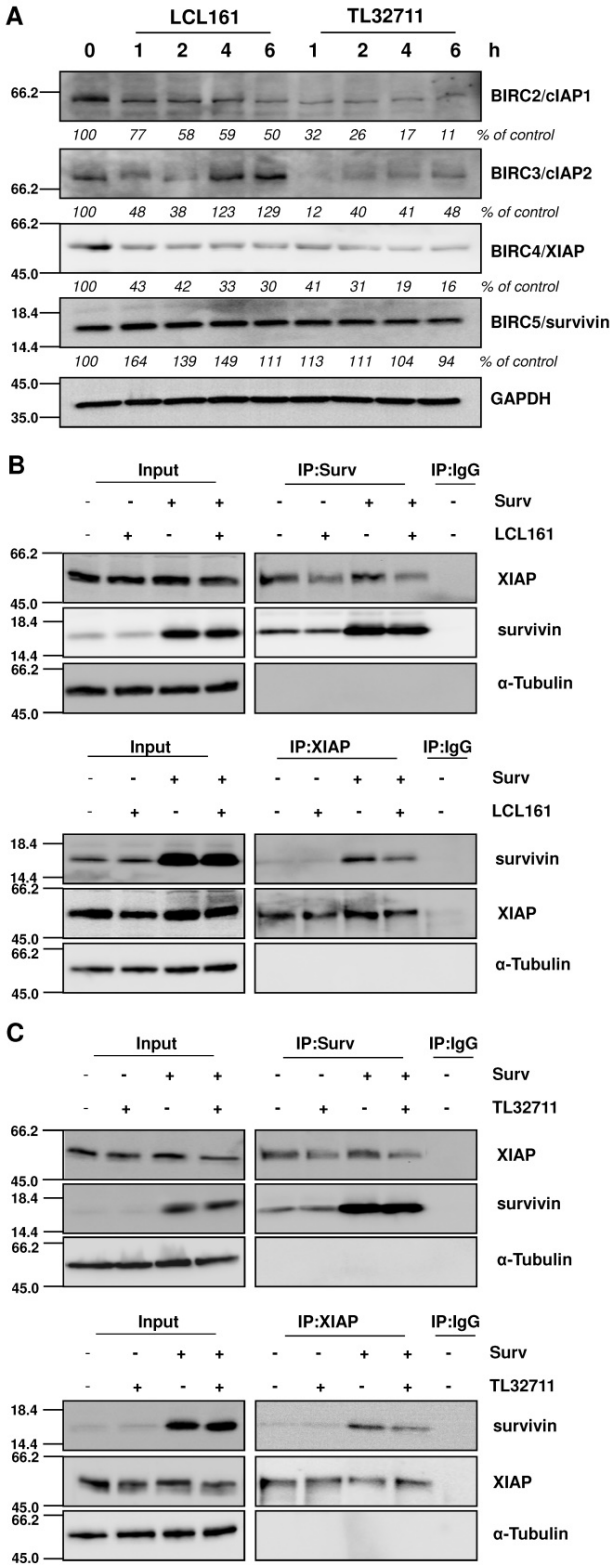
** SMAC-mimetics displace survivin from XIAP.** (**A**) SH-EP cells were treated for the times indicated with 20 µM LCL161 or TL32711 respectively. Cells lysates were subjected to immunoblot analyses for cIAP1, cIAP2, XIAP and survivin. GAPDH served as loading control. SH-EP/Ctr and SH-EP/Surv (Surv) cells were treated with 10 µM LCL161 (**B**) or 10 µM TL32711 (**C**) for 2 hours. Cell lysates were subjected to immunoprecipitation for both anti-survivin and IgG control (upper panel) or anti-XIAP and IgG control (lower panel). Input lysates and precipitates were subjected to immunoblot analyses with antibodies directed against survivin and XIAP. α-Tubulin served as loading control.

Since SMAC-mimetics were designed to specifically induce degradation of those IAP family members that contain a BIR3 domain, we tested the effect of those compounds on the protein levels of XIAP, cIAP1/2 and survivin. As demonstrated in the immunoblot in **Figure 2A**, cIAP1 and XIAP levels continuously decrease, whereas survivin which lacks a BIR3 domain, remains constant or even slightly increases at 20 µM treatments with SMAC-mimetics. For cIAP2 we detected only a transient decline, since its expression levels increased after 4 hours LCL161 treatment and already after 2 hours TL32711 treatment, although others have described a permanent loss of cIAP2 at similar *in vitro* concentrations 32, 33. To further test our hypothesis that mitochondrial fragmentation seen in **Figure 1** results from the disruption of XIAP/survivin complexes and from released survivin, we performed co-immunoprecipitation experiments for survivin and XIAP after LCL161 and TL32711 treatment. Both SMAC-mimetics reduced the amount of XIAP bound to survivin within two hours treatment in SH-EP/Ctr and in SH-EP/Surv cells (**Figure 2B and 2C, upper panels**) and *vice versa,* less survivin was bound to XIAP after SMAC-mimetic treatment (**Figure 2B and 2C lower panels**). Of note, SMAC-mimetic-treatment reduced XIAP-steady state levels which might also contribute to the release of survivin.

**Figure 3:**
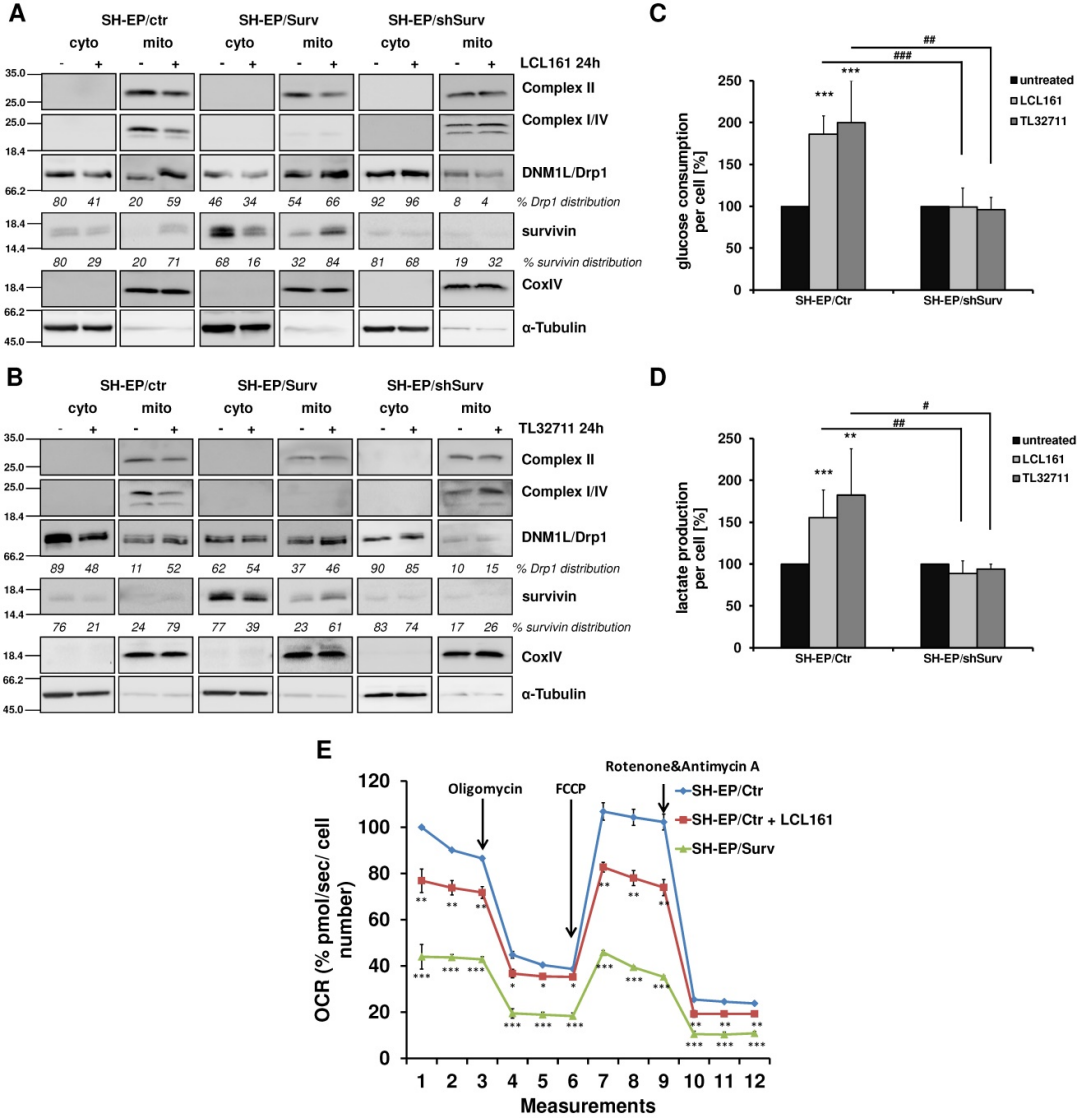
** Mitochondrial reorganization induces a metabolic shift to glycolysis.** SH-EP/Ctr, SH-EP/Surv, and SH-EP/shSurv cells were treated with 20 µM LCL161 (**A**) or 24 µM TL32711 (**B**) for 24 hours. Cytoplasmic and mitochondrial extracts were analyzed for expression of mitochondrial respiration complexes I, II and IV (OXPHOS) as well as DRP1. CoxIV (mitochondrial) and α-Tubulin (cytoplasmic) served as markers for extract purity. Glucose consumption (**C**) and lactate production (**D**) was monitored after 72 hours treatment with low-dose LCL161 (5 µM) or TL32711 (3 µM). Untreated cells were set as 100%. Shown is the mean+SD of three independent experiments. Statistical differences between untreated and treated cells (***P<0.001, **P<0.01) or knock-down and control cells (### P<0.001, ##P<0.01, #P<0.05) were assessed by unpaired t-test. (**E**) Mitochondrial respiration was analyzed in SH-EP cells treated with 5 µM LCL161 for 72 hours using MitoStress Kit (Agilent, Santa Clara, USA) in a Seahorse XFp. SH-EP/Surv cells were used as control. Shown is the mean±SD of four independent experiments. Statistical differences to SH-EP/Ctr cells (***P<0.001, **P<0.01, *P<0.05)

The release of survivin from XIAP/survivin complexes further caused translocation of DRP1 from the cytoplasm to mitochondria and reduction of DRP1 phosphorylation at Ser637 (**Figure 3A and 3B, Supplementary Figure S2E**). The results of subcellular fractionation were confirmed by immunofluorescence staining, where in SMAC-mimetic treated cells DRP1-staining strongly co-localized with CMXRos-stained mitochondria (**Supplemental Figure S2F and S2G**). This was in line with the observed mitochondrial fragmentation in **Figure 1** and **Supplementary Figure S2B** where DRP1 knock-down prevents mitochondrial fragmentation and with previous results that high survivin expression leads to increased mitochondria-associated DRP1^24^. Additionally, LCL161 and TL32711 treatment reduced the expression of respiratory complexes I, II, and IV in SH-EP/Ctr cells, but not in SH-EP/shSurv cells, which is again reflecting the high-survivin-expressing phenotype as published before 24. Since we have seen before that survivin shuts down respiration and leads to dependency on aerobic glycolysis, we tested whether mitochondrial fragmentation by SMAC-mimetics also affects cellular metabolism. Therefore we monitored the glucose amount and the lactate release into the media of SH-EP/Ctr and SH-EP/shSurv cells after treatment with LCL161 (5 µM) and TL32711 (3 µM) for 72 hours. In SH-EP/Ctr cells expressing endogenous levels of survivin, LCL161 and TL32711 cause an increase in glucose consumption and simultaneously an increased lactate release into the media. In SH-EP/ shSurv cells, however, no significant changes in glucose consumption or lactate production were visible (**Figure 3C and 3D**). Our results were further supported by GC/MS-based quantitation of intracellular levels of metabolites belonging to glycolysis and TCA cycle pathways. As shown in **Supplemental Figure S3**, treatment for 72 hours with LCL161 (5 µM) reduces the amount of glucose, citrate, succinate, fumarate, and malate comparable to ectopic survivin expression and suggest that treatment with LCL161 preferentially induces the direct conversion of pyruvate into lactate instead of generating TCA cycle metabolites. To finally test whether SMAC-mimetics affect oxidative phosphorylation (OXPHOS), we measured mitochondrial respiration after LCL161 treatment (5 µM, 72 hours). As shown in **Figure 3E**, treatment with LCL161 significantly reduced oxygen consumption rates (OCR) in SH-EP/Ctr cells to less than 80% compared to untreated controls. As a control, we also measured OCR in SH-EP/Surv cells, which only possessed about 40% mitochondrial respiration ability compared to SH-EP/Ctr cells. This further strengthens our hypothesis, that survivin, released from XIAP is sufficient to induce mitochondrial morphology changes, which affect cellular metabolism.

### Reduction of respiratory complexes hinders ROS production

**Figure 4:**
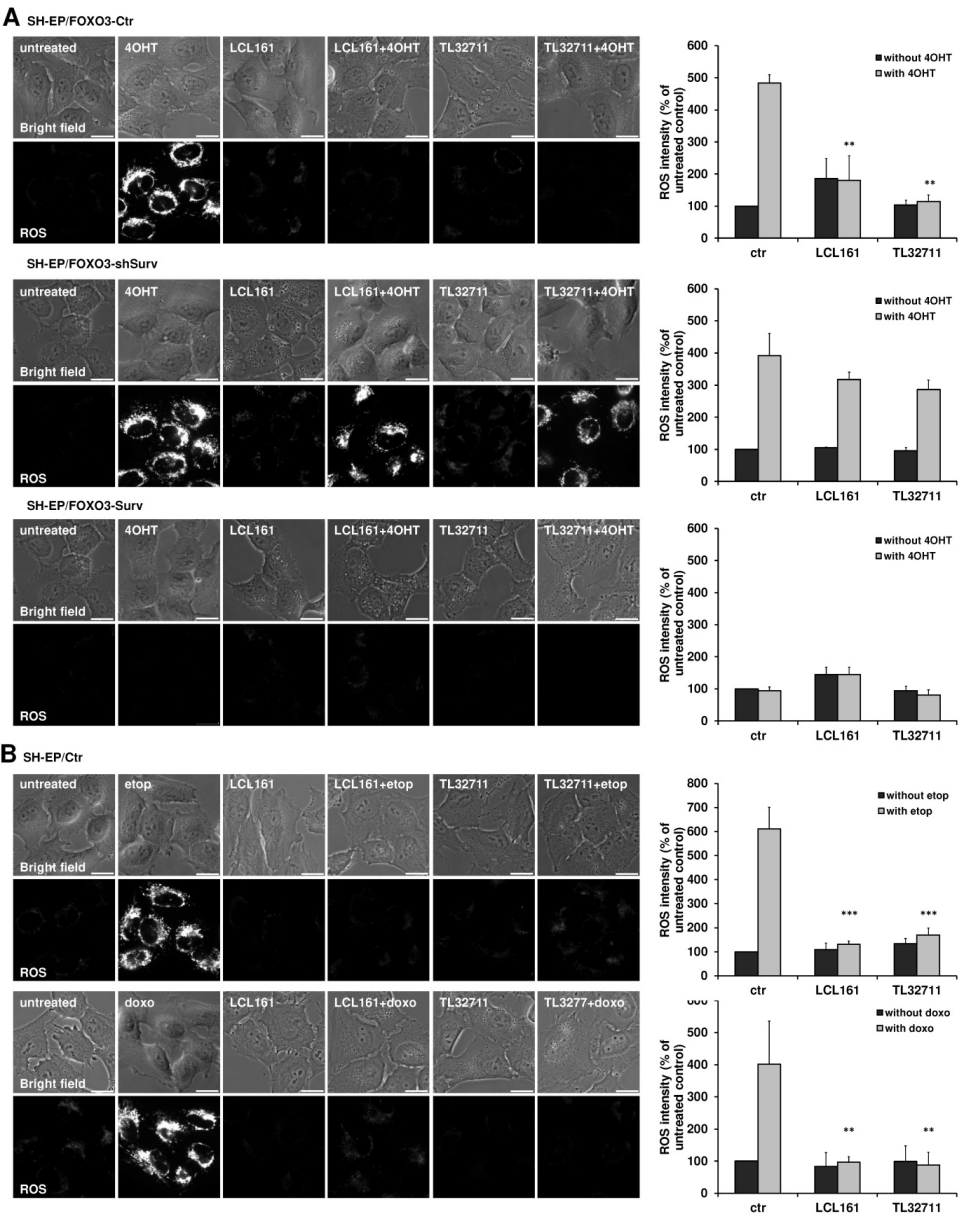
** SMAC-mimetics prevent ROS generation.** (**A**) SH-EP/FOXO3-Ctr, SH-EP/FOXO3-shSurv and SH-EP/FOXO3-Surv cells were treated with 100 nM 4OHT for 4 hours alone or in combination with 10 µM LCL161 or TL32711, respectively (20 hours pre-incubated). (**B**) SH-EP cells were treated with 10 µg/ml etoposide (2 hours) or 0.25 µg/ml doxorubicin (6 hours) alone or in combination with 10 µM LCL161 or TL32711, respectively (20 hours pre-incubated). ROS production was detected by live cell imaging using MitoTracker Red CM-H_2_XROS (500 nM). For the quantification of cellular ROS intensity, the cells of three independent experiments (in each experiment three to four micrographs, more than 30 cells per experiment) were densitometrically analyzed using Axiovert Software (Zeiss, Vienna) and statistical differences were assessed by unpaired t-test (***P<0.001; **P<0.01).

The shift from OXPHOS to glycolysis reduces the amount of ROS which are produced during the electron transfer within the respiratory chain. Since the reduction of complex I, II and IV and the increase in glycolysis suggest an effective shift away from respiration, we tested the ability of LCL161- or TL32711-treated cells to produce ROS. We therefore chose a PKB-independent 4-hydroxy-tamoxifen (4OHT) inducible FOXO3(A3)ER construct, which was already shown to affect mitochondrial respiration and ROS generation *via* BCL2L11/Bim induction 28. To investigate the effect of SMAC-mimetics on ROS production we pre-treated SH-EP/FOXO3-Ctr, SH- EP/FOXO3-shSurv or SH-EP/FOXO3-Surv cells with 10 µM LCL161 or TL32711 for 20 hours to induce mitochondrial changes before we added 100 nM 4OHT for another 4 hours. Activation of FOXO3 by 4OHT leads to a strong accumulation of mitochondrial ROS as visualized by MitoTracker Red staining. This ROS burst was efficiently reduced when the cells were pre-incubated with the SMAC-mimetics (**Figure 4A, upper panel**) although Bim was still induced within these cells (**Supplementary Figure S4A**). As described before, mitochondrial ROS- accumulation by FOXO3 contributes to apoptosis induction - the pre-treatment with LCL161 or TL32711 significantly reduced apoptosis after ectopic FOXO3 activation (**Supplemental Figure S4B**). In survivin knock-down cells, however, neither LCL161 nor TL32711 were able to reduce ROS levels significantly (**Figure 4A, middle**). In cells ectopically expressing survivin, no ROS accumulation was detectable independent of SMAC-mimetic treatment (**Figure 4A, lower panel**).

The ROS-inhibiting function of LCL161 and TL32711 was also visible when the cells were treated with the DNA-damaging chemotherapeutics etoposide and doxorubicin: both drugs failed to induce mitochondrial ROS when the cells were pre-treated with the SMAC-mimetics (**Figure 4B**). This interesting finding uncovers a “dark-side” of SMAC-mimetics in cancer therapy and might explain the failure of phase I and phase II studies on SMAC-mimetic administration over the past years (https://clinicaltrials.gov; NCT02147873 and 17).

### The metabolic shift induced by SMAC-mimetics sensitizes cancer cells to non-toxic glycolysis inhibition

**Figure 5:**
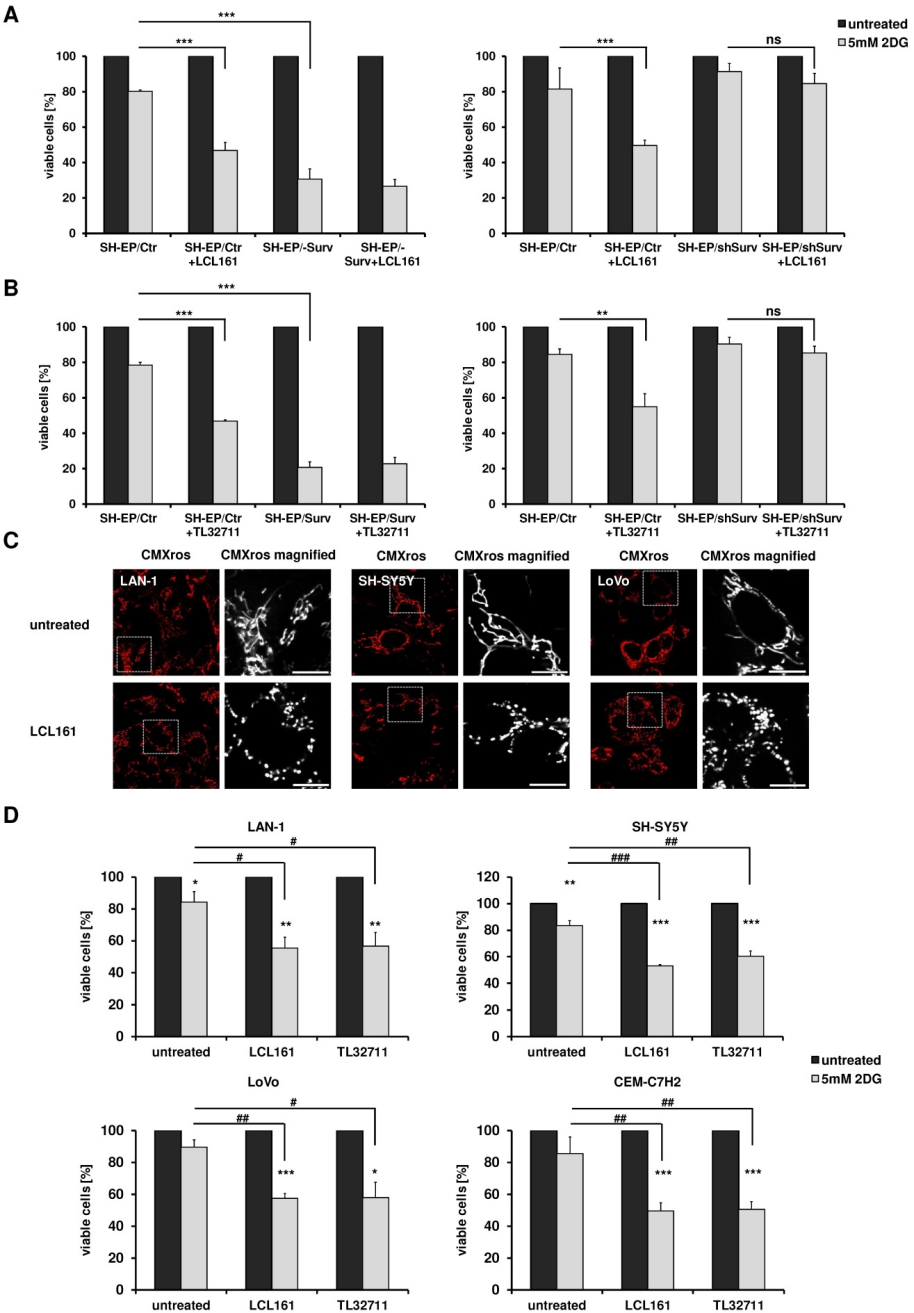
** Mitochondrial re-organization sensitizes cells for glycolysis-inhibition.** SH-EP/Ctr and SH-EP/Surv (left panel) or SH-EP/Ctr and SH-EP/shSurv cells (right panel) were pre-treated for 12 hours with 10 µM LCL161 (**A**) or 12 µM TL32711 (**B**). 5 mM 2DG were added for another 24 hours. Cell viability was assessed by resazurin reduction. Untreated controls/ SMAC-mimetic-treated cells were set as 100%. Shown is the mean+SD of at least three independent experiments. Statistical differences were assessed by unpaired t-test (***P<0.001;**P<0.01). (**C**) Fluorescence analyses of mitochondrial morphology in LAN-1, SH-SY5Y and LoVo cells after treatment with 10 µM LCL161 for 24 hours. Mitochondria were stained with 300 nM CMXRos. Images were collected with a 63x oil objective in an Axiovert200M microscope equipped with an ApoTome.2 system. (**D**) LAN-1, SH-SY5Y, LoVo, and CEM-C7H2 cells were pre-treated with LCL161 (6 µM) or TL32711 (12 µM) for 12 hours before 5 mM 2DG was added for additional 24 hours. Cell viability was measured by resazurin reduction. Shown is the mean+SD of three independent experiments. Statistical differences were assessed by unpaired t-test (***P<0.001, **P<0.01, *P<0.05, ###P<0.001, ##P<0.01, #P<0.05).

Our results suggest that SMAC-mimetics displace survivin from XIAP, which leads to DRP1 recruitment to mitochondria, mitochondrial fragmentation, respiration reduction and an increase in glycolysis. Since we demonstrated before that highly glycolytic tumors with increased survivin levels can be efficiently targeted by glycolysis- inhibition 24, 25, we now tested, whether the observed changes in metabolism induced by SMAC-mimetics are sufficient to sensitize cells for glycolysis inhibition. We therefore pre-treated SH-EP/Ctr, SH-EP/Surv and SH-EP/shSurv cells for 12 hours with LCL161 (**Figure 5A**, 10 µM) or TL32711 (**Figure 5B**, 12 µM) before we added 5 mM 2DG for another 24 hours. After treatment cell viability was analyzed by resazurin reduction. As demonstrated in **Figure 5A** and B, 2DG-treatment alone hat only a minor effect by reducing cell viability to 80-90% compared to untreated controls. Co-administration of LCL161 or TL32711, however, significantly reduced viability below 50%. In SH-EP/Surv cells, which are mainly glycolytic, 2DG-treatment alone reduced viability below 30% and SMAC-mimetics had no additional sensitizing effect. In SH-EP/shSurv cells (**Figure 5A and 5B, right panel**) both SMAC-mimetics failed to reduce viability after 2DG-treatment, suggesting again that survivin levels are critical for shifting cells into glycolysis. As a control, we also lowered survivin levels by addition of YM155, a compound that inhibits survivin and was recently shown to also enhance degradation of survivin 9, 34. When cells were treated with 5 nM YM155 and LCL161, LCL161 failed to sensitize these cells for 2DG-treatment (**Supplemental Figure S5**), demonstrating again, that unbound survivin is necessary for shifting cells towards glycolysis.

To further explore the specificity of the observed sensitization to 2DG by co-treatment with SMAC- mimetics, we analyzed the effect of LCL161 on two other neuroblastoma cell lines which do not carry an 17q amplification, but are derived from biopsies of patients with stage M (old classification stage 4 35) and further differ in the N-MYC status 36. Also in these two neuroblastoma cell lines (SH-SY5Y and LAN-1) LCL161 treatment significantly reduced mitochondrial connectivity (**Figure 5C**) and LCL161 or TL32711 pre-treatment sensitized these cells to 2DG (**Figure 5D**). This effect was not only restricted to neuroblastoma cells, but also observed in the colorectal adenocarcinoma cell line LoVo or in the leukemia cell line CEM-C7H2 (5C/D). As shown in Supplemental **Figure S6C**, within these cell lines we observed a moderate survivin/XIAP ratio, suggesting that not only absolute surviving or XIAP expression levels, but the ratio between XIAP and survivin is critical for the metabolic phenotype. Cell lines with amplified chromosome 17q (STA-NB15) or ectopic expression of surviving (SH-EP/Surv) possess an increased survivin/XIAP ratio and are *per se* highly glycolytic 24, 25. In contrast, in the mouse neuroblastoma cell line NxS2 which already has an largely fragmented mitochondrial phenotype, SMAC-mimetics hat no sensitizing effect to 2DG treatment, but these cells already showed reduced viability after 2DG single treatment comparable to SH-EP/Surv cells (**Supplemental Figure S6A and S6B**).

**Figure 6:**
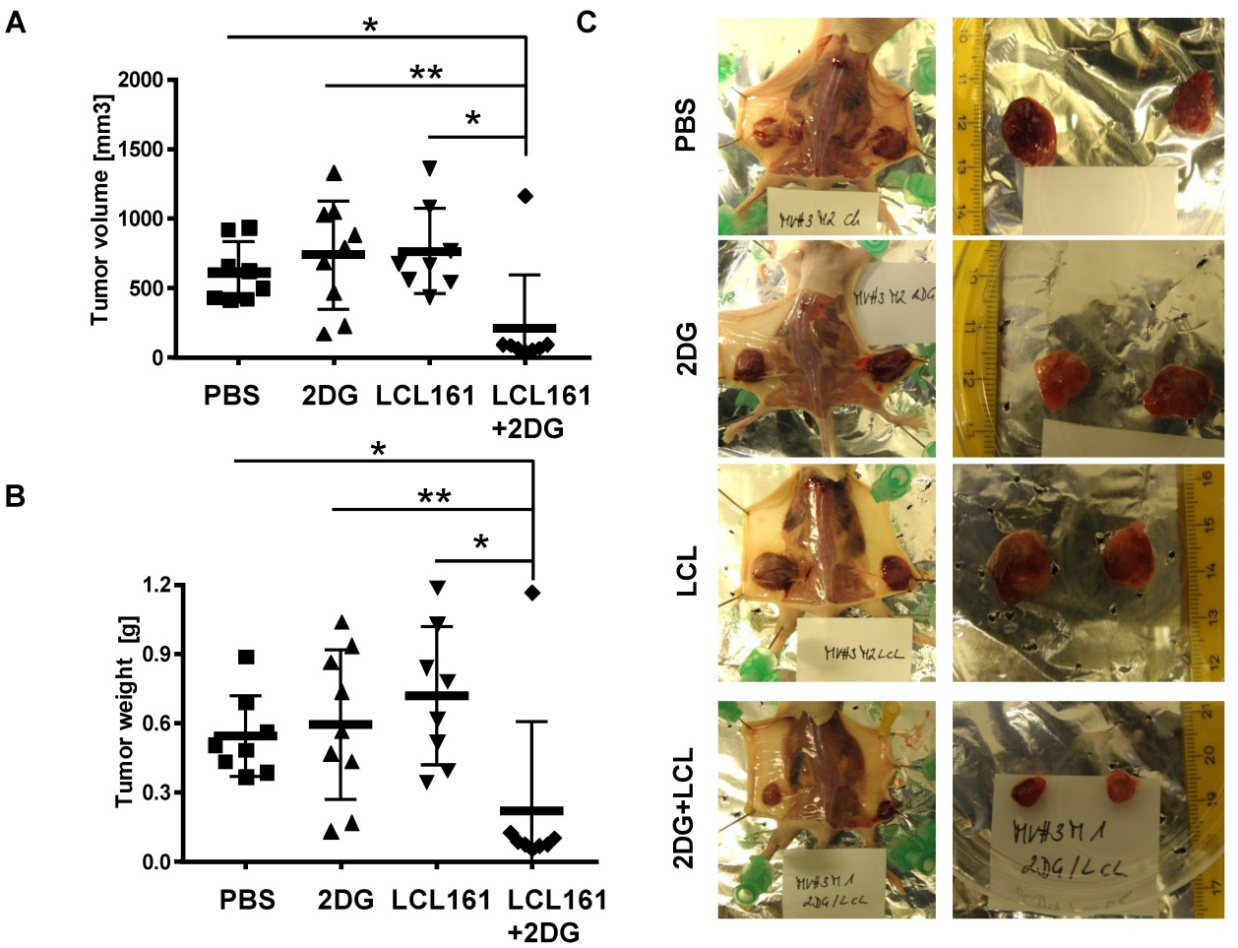
** 2DG and LCL161 prevent tumor growth *in vivo*.** 2x10^7^ LAN-1 cells were injected into the flanks of Balb c nu/nu mice. After tumors were palpable (approximately 50 mm^3^) mice received 5 mg/0.2 ml 2DG, 4 mg/kg LCL161 or a combination of both in solvent (PBS/EtOH). One group was treated with solvent only. After four weeks mice (6 mice per group) were sacrificed and tumor weight (**B**) and volume (**A**) were calculated (volume = (length * width^2^)/2). Statistical differences of tumor volumes or tumor weight were assessed using Mann-Whitney-U-test (**P<0.01, *P<0.05). (**C**) Representative images of tumors for each treatment group.

Finally, we tested whether the observed reduction in cell viability by co-administration of SMAC-mimetics and 2DG is also relevant under physiological conditions with glucose *ad libitium.* We injected LAN-1 cells which were shown to be tumorigenic in mice 37, into the flanks of Balb c nu/nu mice to study the effects of combined SMAC-mimetic / 2DG therapy *in vivo* on highly malignant neuroblastoma tumors. After tumors were palpable (approximately 50 mm^3^), the mice were randomly divided into four groups and treated three times a week for three weeks: one group was treated i.p. with 5 mg 2DG, the second group received 4 mg/kg LCL161, the third group a combination of both and the control group was treated with carrier only. Tumor volume (**Figure 6A** and representative pictures in **Figure 6C**) and tumor weight (**Figure 6B**) was significantly reduced in LAN-1-derived tumors treated with LCL161 and 2DG after 3 weeks, whereas neither LCL161 alone nor 2DG alone reduced tumor growth. Except for one tumor, all LAN-1 derived tumors remained smaller than 100 mm^3^. This suggests that the combinational therapy of LCL161 and 2DG is a highly effective treatment option for neuroblastoma and potentially other tumors, even if the tumor cells express only moderate levels of survivin.

## Discussion

Apart from resistance against chemotherapeutic agents and relapsed tumors, the risk of secondary malignancies caused by initial chemotherapy is especially high for children facing extensive chemotherapy 38. Therefore, cancer specific, non-genotoxic and patient oriented “precision medicine” have come into the focus of targeting cancers during the last years 39, 40. One class of these cancer specific agents which have entered preclinical studies are so called SMAC-mimetics which are designed to inhibit the inhibitor-of-apoptosis protein members BIRC2/ cIAP1, BIRC3/cIAP2 and BIRC4/XIAP 17. These agents are currently used as mono-, and even more effective, in combination-therapy to override cell-death inhibitory signaling. Although these substances are now tested for several years, there is still debate about their efficacy, their interplay with several pathways and their activity in preclinical studies 41. We previously identified another IAP member, BIRC5/survivin as critical factor in shifting mitochondrial energy production from OXPHOS to aerobic glycolysis by regulating mitochondrial connectivity in high-stage neuroblastoma 24, 25. Since survivin shares a BIR-domain with the other IAP members and is thought to be stabilized by XIAP 10, 42, we tested whether SMAC-mimetics might also affect the mitochondrial morphology changes induced by survivin. To our surprise different SMAC- mimetics were ineffective to reverse the survivin- induced phenotype (**Figure 1B**), but induced mitochondrial fragmentation in SH-EP/Ctr cells (**Figure 1A**) without inducing cell death (**Supplemental Figure 1**). This suggests that SMAC-mimetic-induced degradation of XIAP does not destabilize survivin. The observed effects on mitochondrial morphology were dependent on survivin and DRP1 expression levels since knock-down of either survivin or DRP1 reverses the fragmented phenotype (**Figure 1C, Supplemental Figure S2A and S2B**). The knock- down of XIAP by short-hairpin RNA also increased mitochondrial fragmentation (**Figure 1E**) suggesting that the disruption of XIAP-survivin complexes by SMAC-mimetics either by steric inhibition or degradation of XIAP (**Figure 2B and 2C**) triggers mitochondrial fragmentation *via* unbound survivin. The N-terminal mitochondrial localization signal of survivin 43 might become exposed only in these survivin molecules, which induces their mitochondrial localization. This disruption of XIAP/survivin complexes was accompanied by recruitment of DRP1 from the cytoplasm to mitochondria as well as a reduction of mitochondrial respiration complexes I and IV in SH-EP/Ctr cells to the same amount as in cells with moderate ectopic survivin expression (**Figure 3A and 3B**). Consistent with our results from neuroblastoma cells with amplification of 17q or ectopic expression of survivin, which leads to an increase in aerobic glycolysis, also the treatment with SMAC-mimetics increases the glucose consumption and lactate generation in SH-EP/Ctr cells, but not in SH-EP/shSurv cells (**Figure 3C and 3D**). In addition, ROS generation, induced by the chemotherapeutics doxorubicin, etoposide, or by activation of a 4OHT-inducible FOXO3-allele 28 was inhibited by pre-treatment of LCL161 or TL32711, again comparable to ectopically expressed survivin (**Figure 4** and 24). We have shown before that FOXO3-induced ROS accumulation depends on the induction of the BH3-only protein BCL2L11/Bim 28, but SMAC-mimetics had no effect on transcriptional regulation of Bim (**Supplementary Figure S4A**). Although it was reported that high ROS levels may repress survivin mRNA 44, in case of SMAC- mimetic treatment the lack of ROS-accumulation most likely results from survivin-translocation to mitochondria, mitochondrial fission and downregulation of respiratory complex I 24 as demonstrated in **Figure 3A** and **Figure 3B**. The combined data suggest that SMAC-mimetics cause XIAP degradation and disruption of XIAP/survivin complexes, which in turn releases sequestered survivin, triggers mitochondrial fragmentation by translocation of DRP1 to mitochondria and shifts energy metabolism to aerobic glycolysis. Since aerobic glycolysis correlates with chemoresistance and survivin is frequently expressed in tumor tissue, this might explain why many clinical trials on SMAC-mimetics failed. *Via* this mechanism survivin which is not targeted by SMAC-mimetics (**Figure 2A**) shifts the cells into an even more resistant phenotype that is comparable to cells carrying an amplification of survivin 25. This unwanted and potentially detrimental side-effect of SMAC-mimetics might increase resistance against chemotherapeutic agents that involve ROS-generation at mitochondria for cell death induction. As we recently identified glycolysis- inhibitors as regulators of survivin expression 24, 25 and cancer cells with elevated survivin are strongly glycolytic, we speculated that a combination of SMAC-mimetics with glycolysis-inhibitors might overcome the survivin- induced phenotype. As shown in **Figure 5** the combination of the SMAC-mimetics LCL161 or TL32711 with the 2DG indeed markedly reduced the viability of SH-EP/Ctr cells from over 80% with 2DG alone to 40%. As for mitochondrial fragmentation (**Figure 1**) this effect was dependent on survivin expression, since knock-down of survivin abolished the sensitizing effect (**Figure 5A and 5B, right panel**). Additionally, our results were not limited to neuroblastoma cells, but were also observed in other cancer cell lines like colon cancer or leukemia cells (**Figure 5C and 5D**) and also exerted a highly significant *in vivo* effect in a xenograft transplantation mouse model. This is in line with some reports that simultaneously targeted XIAP and survivin and showed an increased therapy efficacy and reduced tumors growth 45-47. Therefore, analyses of the XIAP/survivin ratio might be useful as a diagnostic tool to predict the efficacy of SMAC-mimetics. Targeting survivin levels *via* glycolysis-inhibitors like 2DG, which are well tolerated and show no severe side effects, offers an attractive, non-genotoxic treatment strategy to increase the efficacy of SMAC-mimetics.

## Supplementary Material

Supplementary figures.

## Figures and Tables

**Figure 7:**
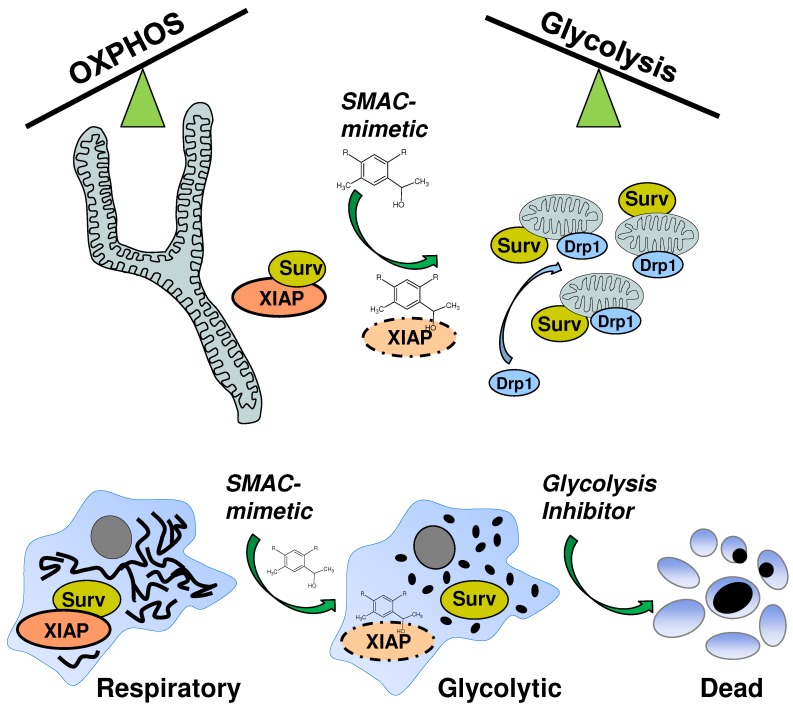
SMAC-mimetic-induced disruption of XIAP/survivin complexes leads to mitochondrial fission via Drp1, shifts cancer cells into a highly glycolytic state and sensitizes them for glycolysis-inhibitor treatment.

## Abbreviations

2DG: 2-deoxy-D-glucose
4OHT: 4-hydroxytamoxifen
BCL2L1/BIM: BCL2-like protein 11 (more commonly used name Bim)
BIR: baculoviral IAP repeat
BIRC4: baculoviral IAP repeat-containing protein 4
BIRC5: baculoviral IAP repeat-containing protein 5
cIAP1: cellular inhibitor of apoptosis protein 1
cIAP2: cellular inhibitor of apoptosis protein 2
DNML1/DRP1: dynamin-1-like protein (HUGO name for more commonly used DRP1)
FACS: fluorescence activated cell sorting
FOXO3: forkhead- box protein O3
IAP: inhibitor of apoptosis protein
OXPHOS: oxidative phosphorylation
OCR: oxygen consumption rates
PI: propidium iodide
ROS: reactive oxygen species
SMAC: second mitochondria- derived activator of caspases
TCA: tricarboxylic acid cycle
XIAP: X-linked inhibitor of apoptosis protein
