# Supplementary material for: Modulation of Respiration and Mitochondrial Dynamics by SMAC-Mimetics for Combination Therapy in Chemoresistant Cancer
Source: Theranostics. 2019 Jul 9;9(17):4909–22. doi: 10.7150/thno.33758 (PMC6691393; doi:10.7150/thno.33758)
Supplement: Supplementary file 1 — Supplementary figures. [file thnov09p4909s1.pdf]

Figure S1

A)

B)

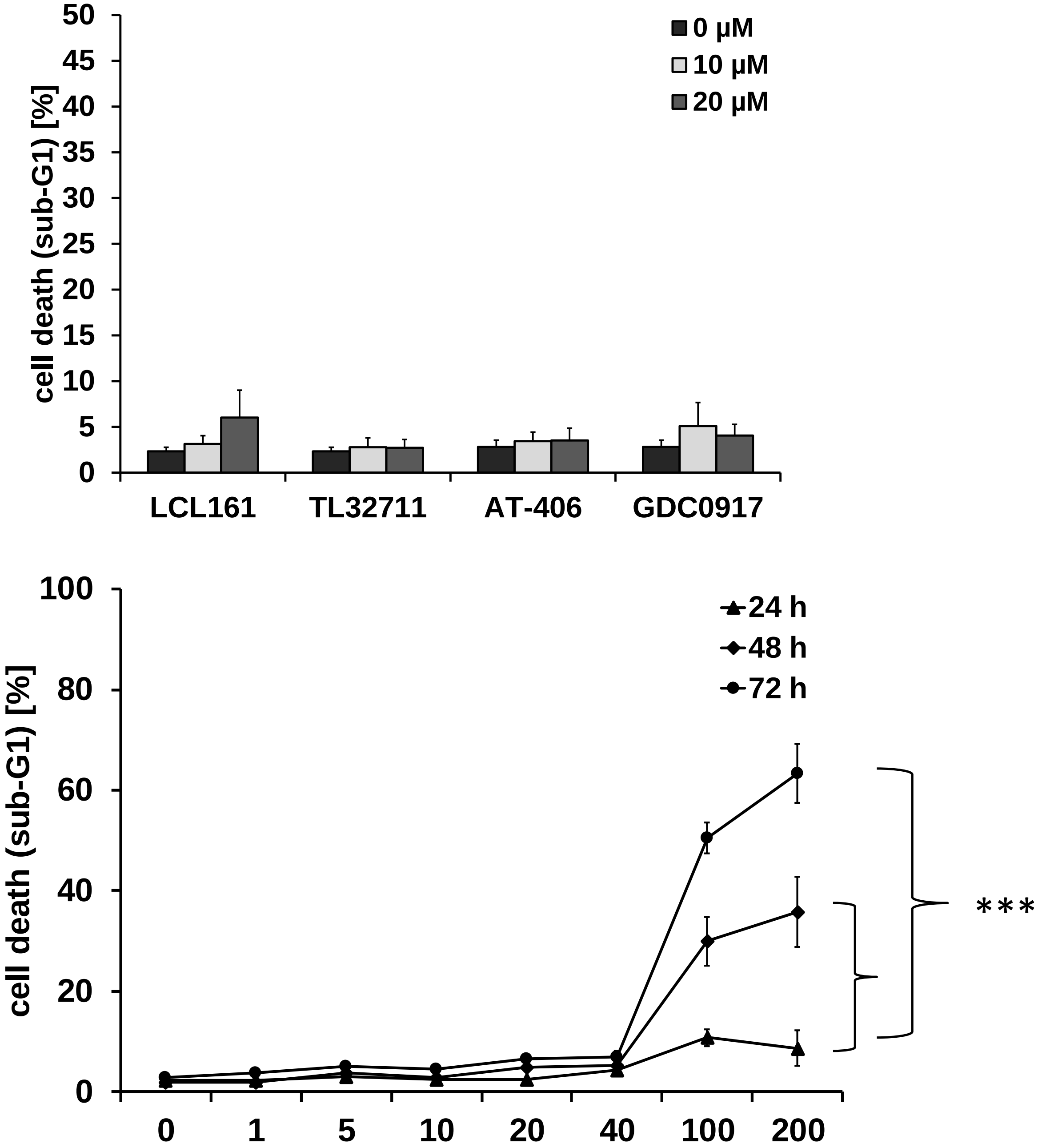

Figure S1: (A) SH-EP/Ctr cells were treated with 10 and 20 µM of the indicated Smac mimetics for 48 hours. (B) SH-EP cells were treated with increasing doses of LCL161 (1-200 µM) for 24, 48, and 72h. Cell death was analyzed by PI-FACS analyses. Shown is the mean of four independent experiments. ***p<0.001 (unpaired t-test).

Figure S1

C)

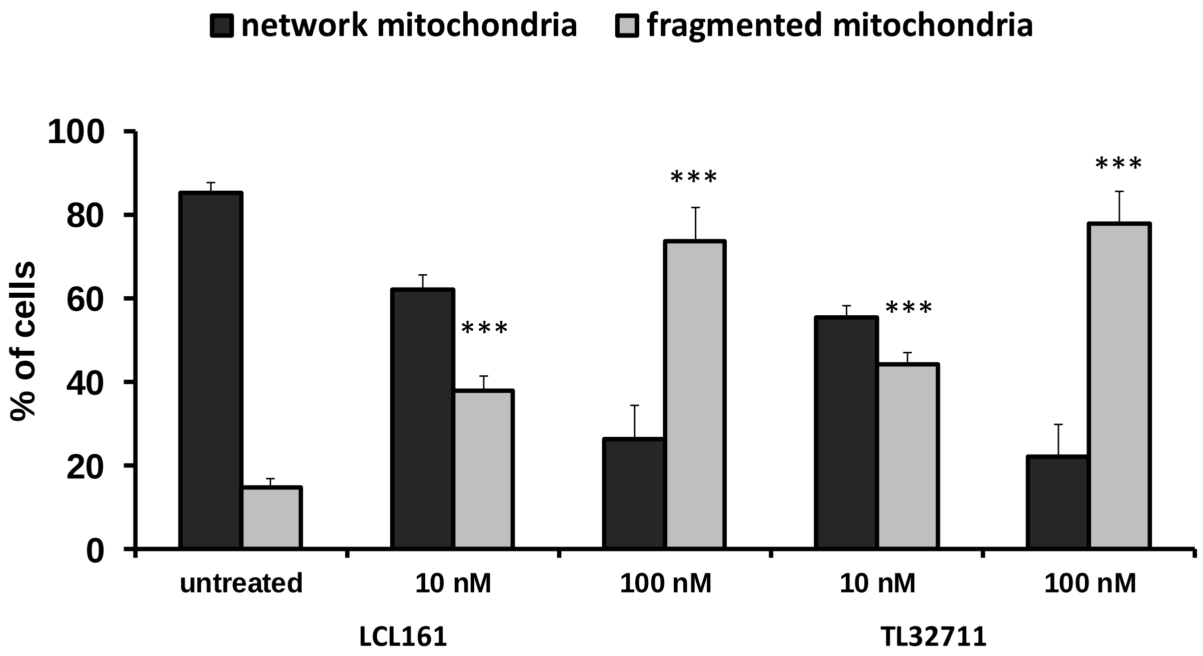

D)

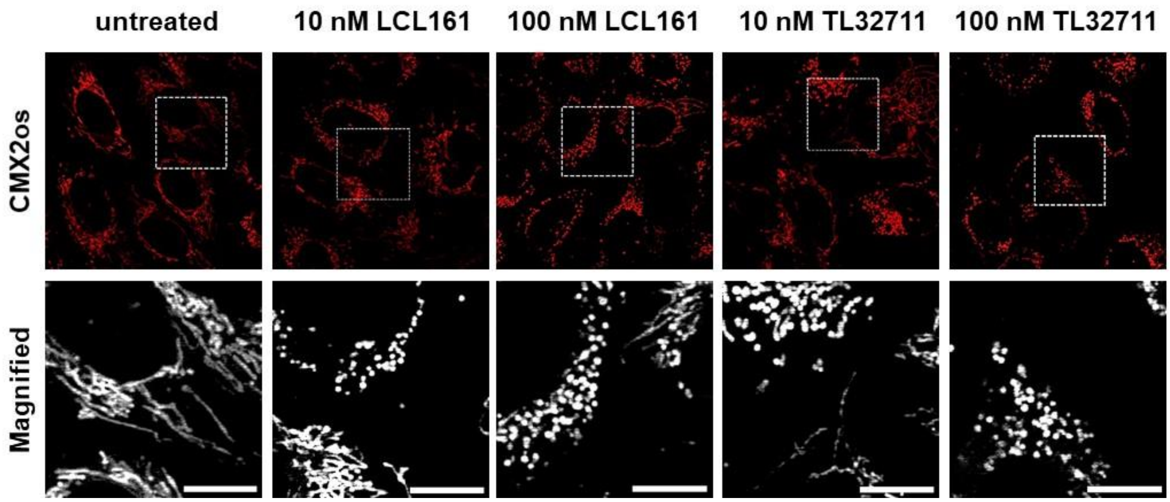

Figure S1: SH-EP/Ctr cells were seeded on glass slides and treated for 72 hours with 10 and 100 nM LCL161 or TL32711. Media was exchanged every 12 hours. Mitochondria were stained with 300 nM CMXRos and analyzed with a 63x oil objective in an Axiovert200M microscope equipped with an ApoTome.2 system. Shown are representative images (D). Bar is 10 µm. For quantification (C), at least 60 cells from three independent experiments were analyzed for mitochondrial morphology. ***P<0.001.

Figure S2

A)

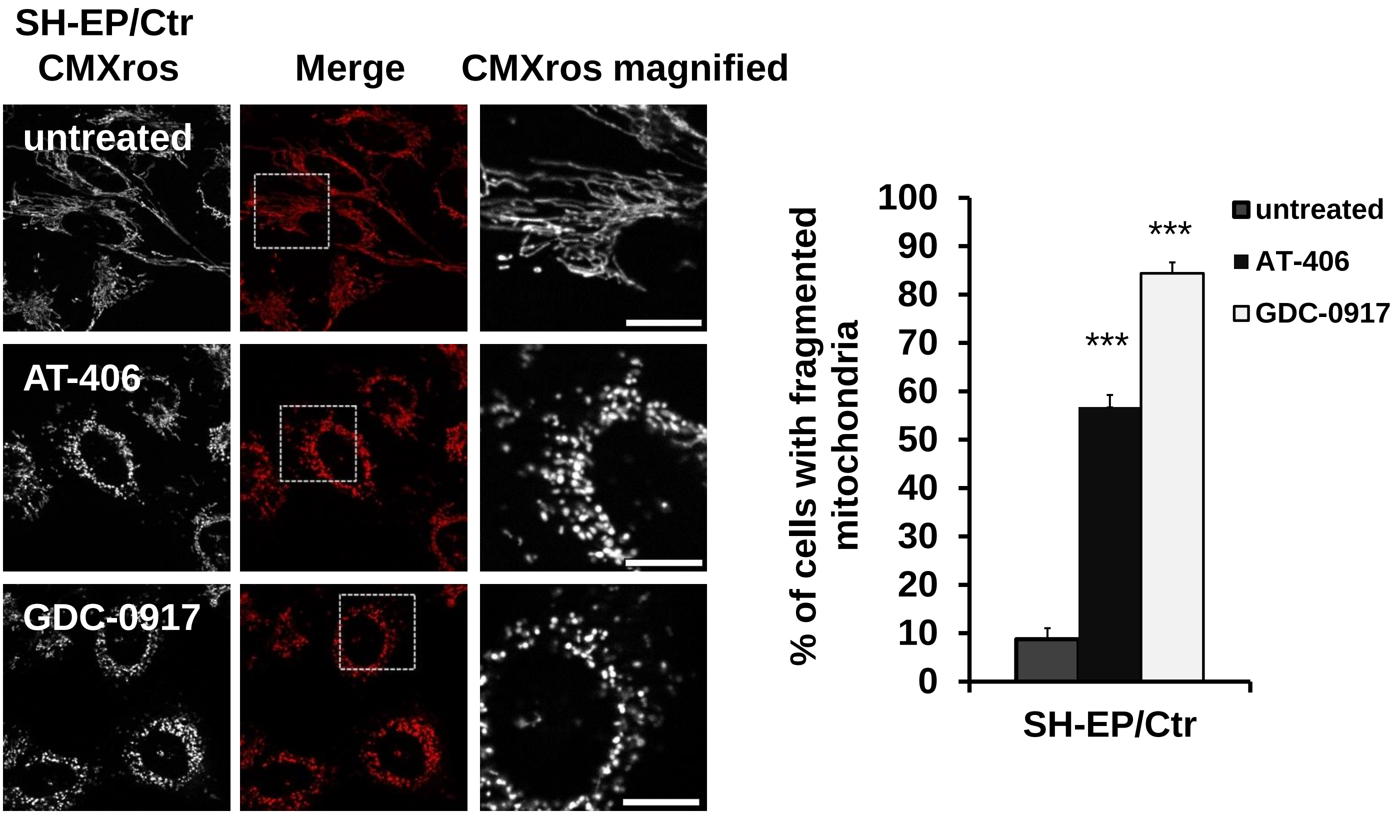
SH-EP/Ctr
CMXros
Merge
CMXros magnified
untreated
AT-406
GDC-0917
% of cells with fragmented mitochondria
100
90
80
70
60
50
40
30
20
10
0
***
***
untreated
AT-406
GDC-0917
SH-EP/Ctr

B)

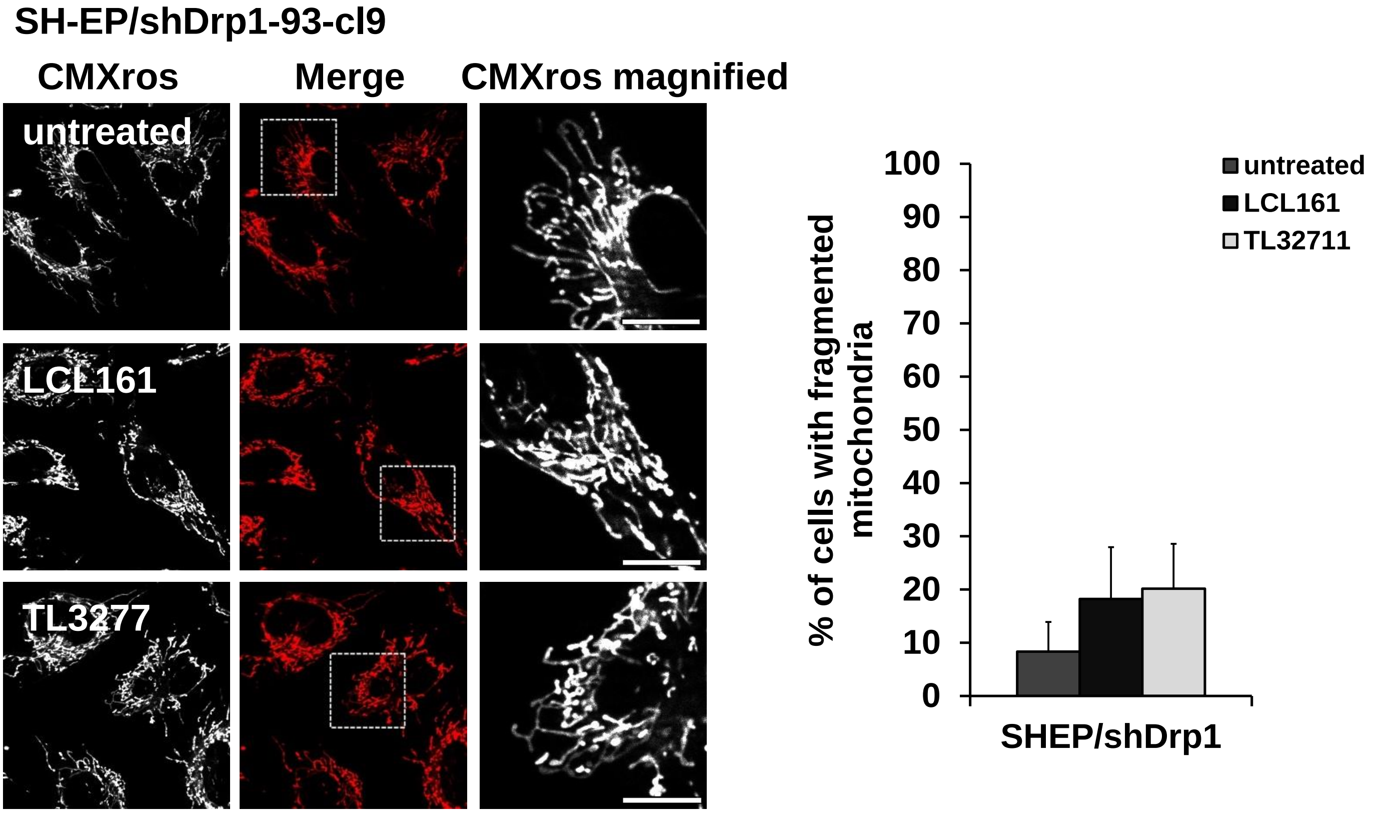
SH-EP/shDrp1-93-cl9
CMXros
Merge
CMXros magnified
untreated
LCL161
TL3277
% of cells with fragmented mitochondria
100
90
80
70
60
50
40
30
20
10
0
untreated
LCL161
TL32711
SHEP/shDrp1

Figure S2

C)

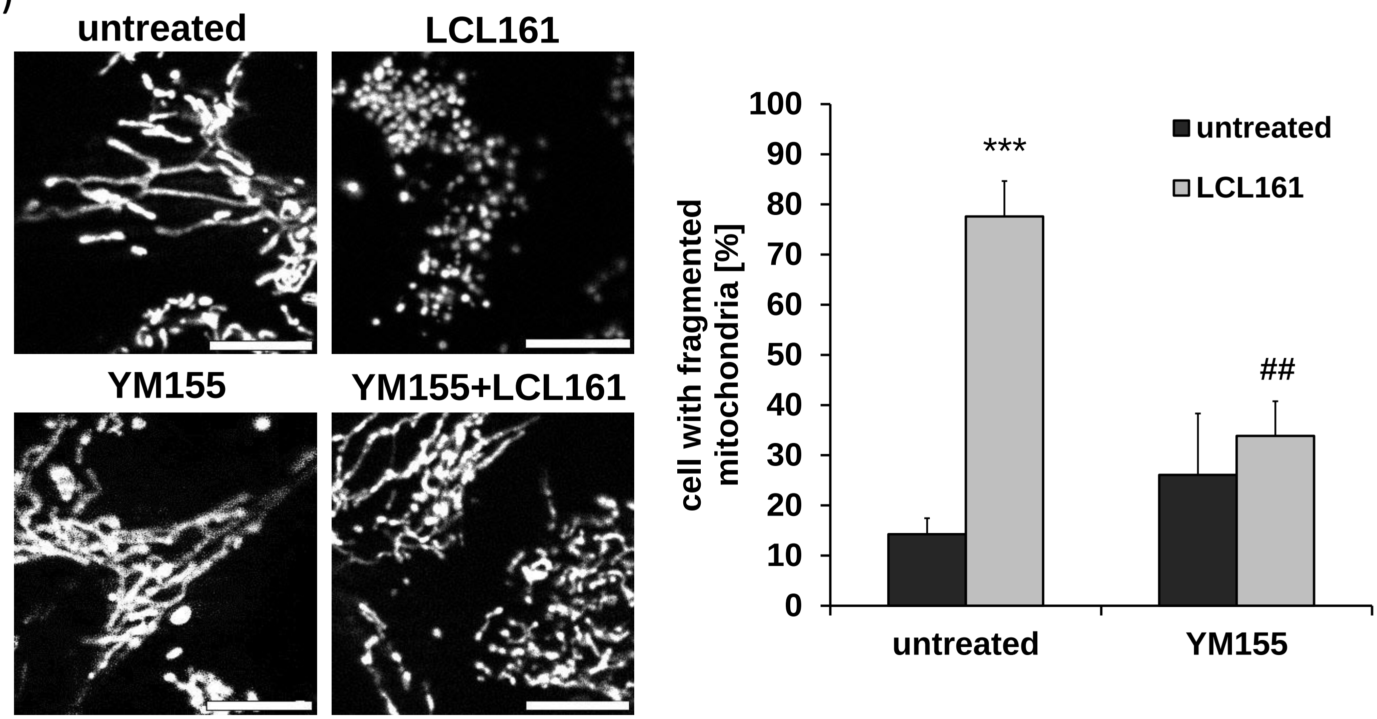

D)

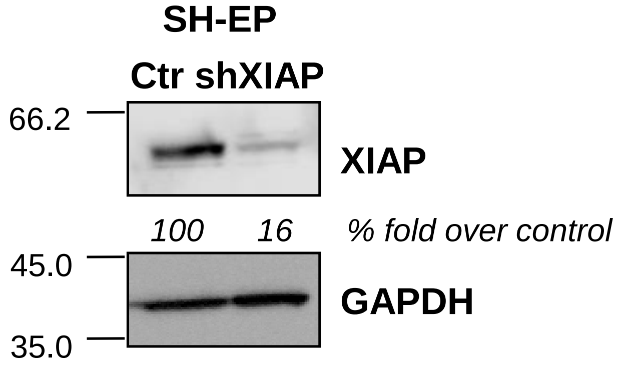

E)

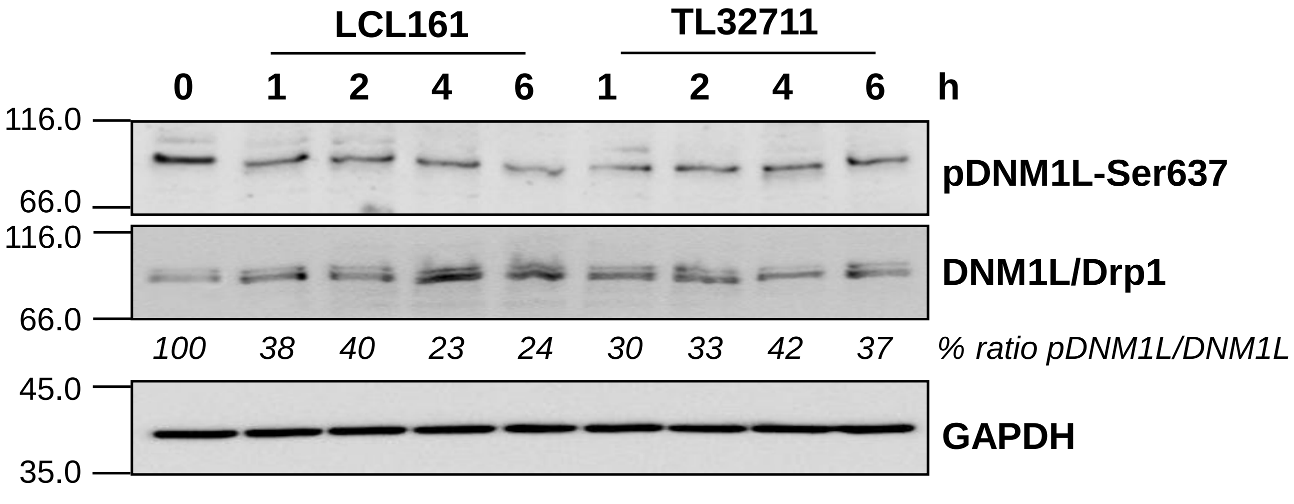

Figure S2

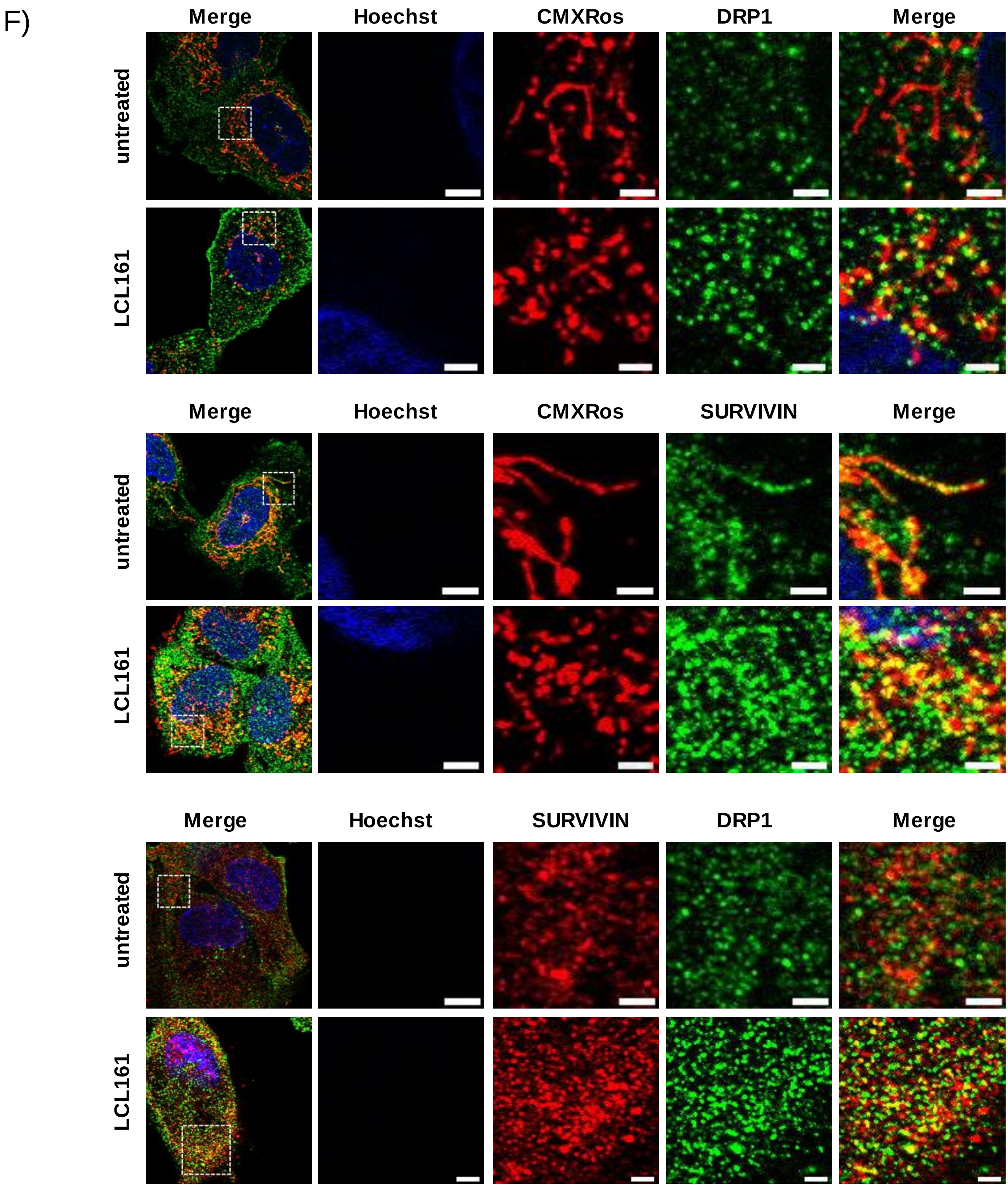

F)
Merge
Hoechst
CMXRos
DRP1
Merge
untreated
LCL161
Merge
Hoechst
CMXRos
SURVIVIN
Merge
untreated
LCL161
Merge
Hoechst
SURVIVIN
DRP1
Merge
untreated
LCL161

Figure S2

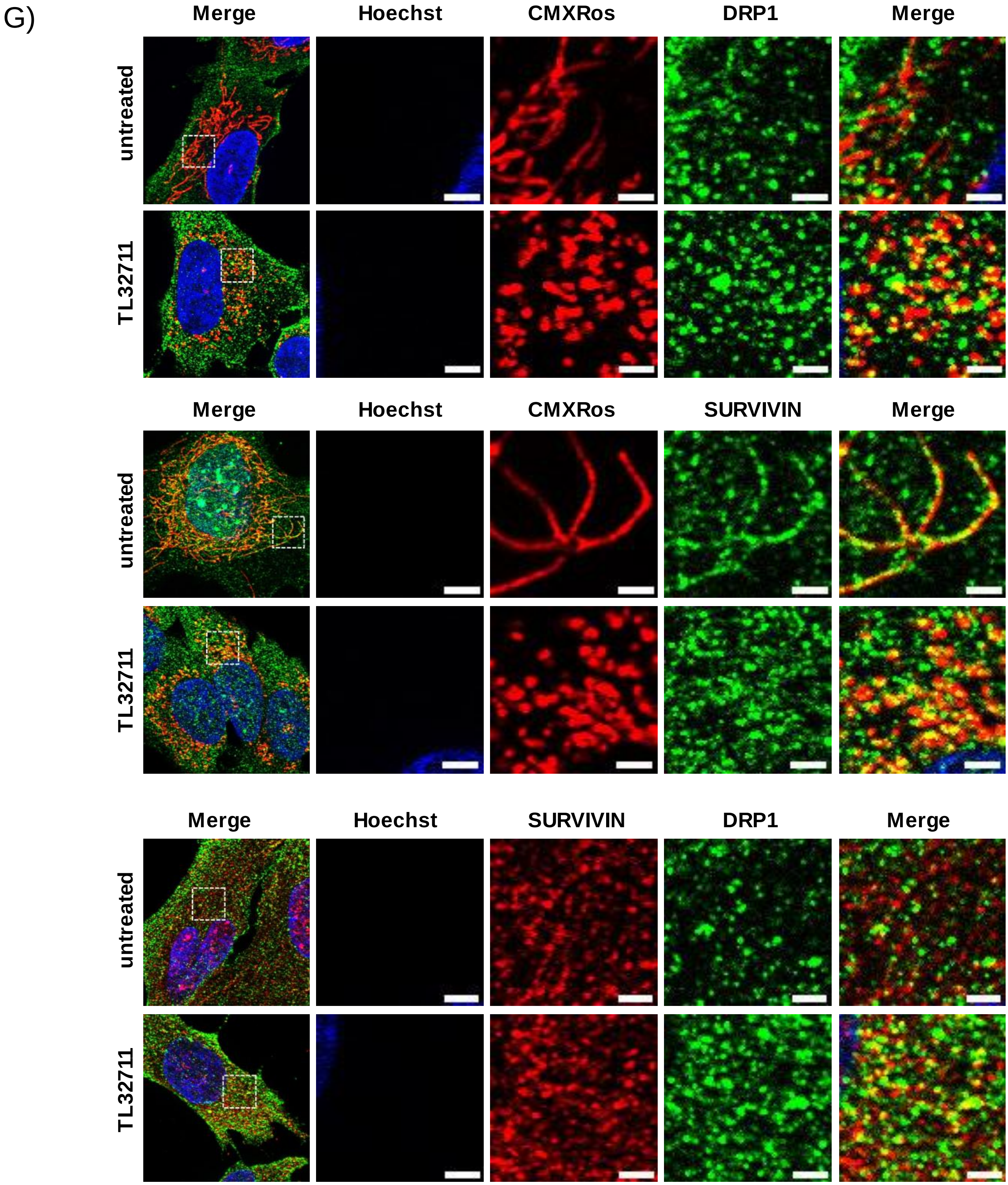
G)
Merge
Hoechst
CMXRos
DRP1
Merge
untreated
TL32711
Merge
Hoechst
CMXRos
SURVIVIN
Merge
untreated
TL32711
Merge
Hoechst
SURVIVIN
DRP1
Merge
untreated
TL32711

Figure S2

Figure S2: (A) SH-EP/Ctr cells were treated for 24 hours with 10 µM AT-406 or GDC-0917. (B) SH-EP/shDrp1-93 cl9 cells were treated for 24 hours with 10 µM LCL161 or TL32711. (C) SH-EP/Surv cells were pre-treated with 10 nM YM155 for 2 hours before 10 µM LCL161 were added for additional 24 hours. Mitochondria were visualized by CMXRos-staining (300 nM) in an Axiovert 200 Zeiss microscope equipped with an ApoTome.2. Bar is 10 µm. For quantification at least 50 cells from three independent experiments were analyzed for mitochondrial morphology. ***p<0.001 between AT-406 or GDC 0917-treatment and untreated; ##p<0.01 between LCL161 treatment and LCL161/YM155 treatment. (D) Knock-down of XIAP was verified by immunoblot. (E) Densitometry of pDrp1-Ser637 and DNM1L-total was measured using LabWorks software. Immunofluorescence staining of Drp1 (upper panel), survivin (middle panel) or Drp1 and survivin (lower panel) with or without 24 hours 10 µM LCL161 (F) or TL32711 (G). For single stainings mitochondria were pre-stained before fixation for 1 h with 300 nM CMXRos, Alexa-488 conjugated secondary antibodies were used for visualization of either Drp1 or survivin. For co-staining Drp1 was visualized with goat anti-mouse Alexa-488, survivin with goat anti-rabbit Alexa-568 (both Thermo Fisher).

Figure S3

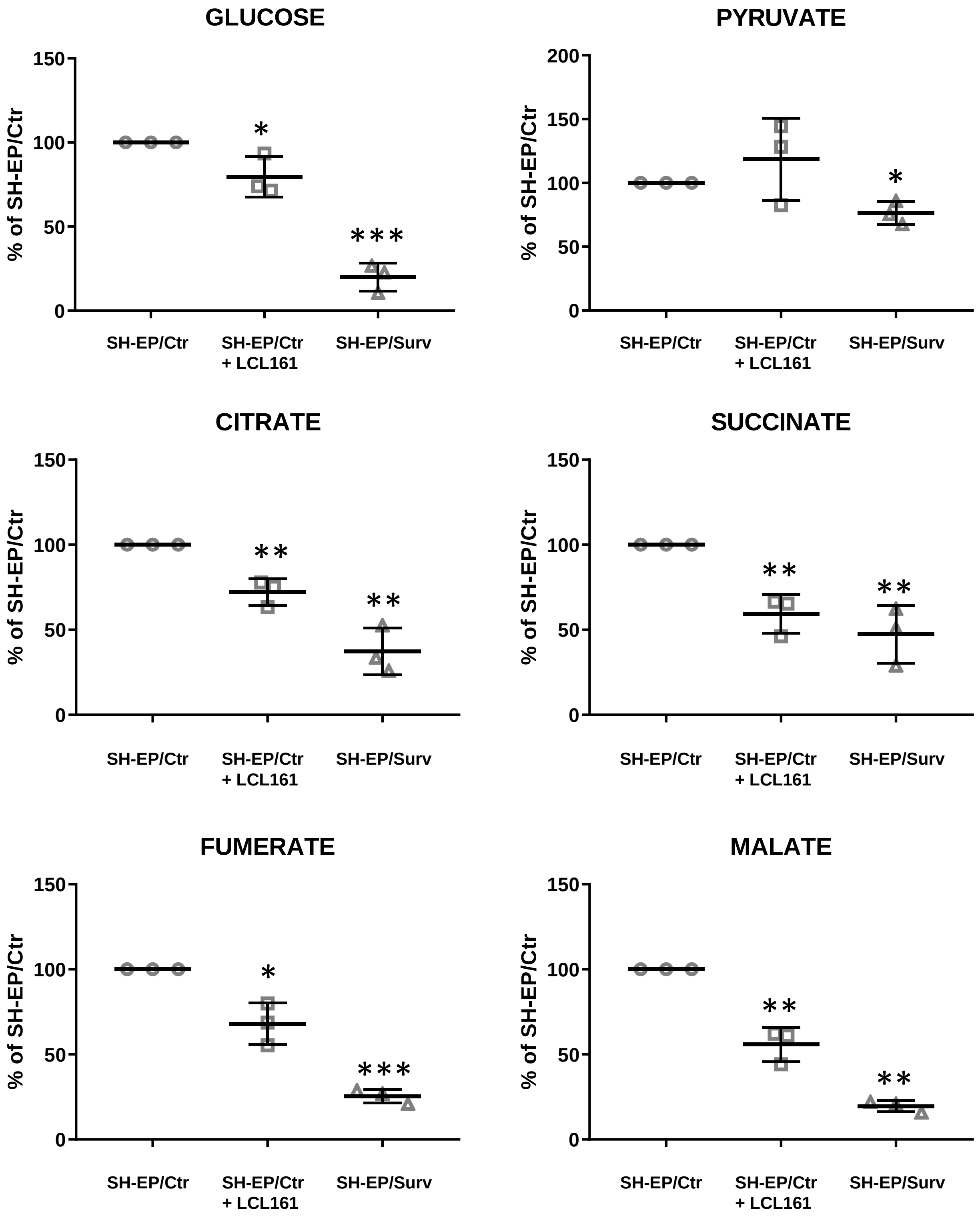

Figure S3: Results of GC-MS of glycolysis and TCA cycle metabolites using methanolic standard solutions of the targeted compounds and nonadecanoic acid as internal standard for calibration. SH-EP/Ctr cells and SH-EP/Surv cells were seeded into cell culture dishes and treated for 72 hours with 5 µm LCL161. $8*10^7$ cells /ml were lysed and subjected to GC-MS. Shown are the absolute means ±SD of three independent experiments. ***P<0.001, **P<0.01, *P<0.05 (unpaired t-test).

Figure S4

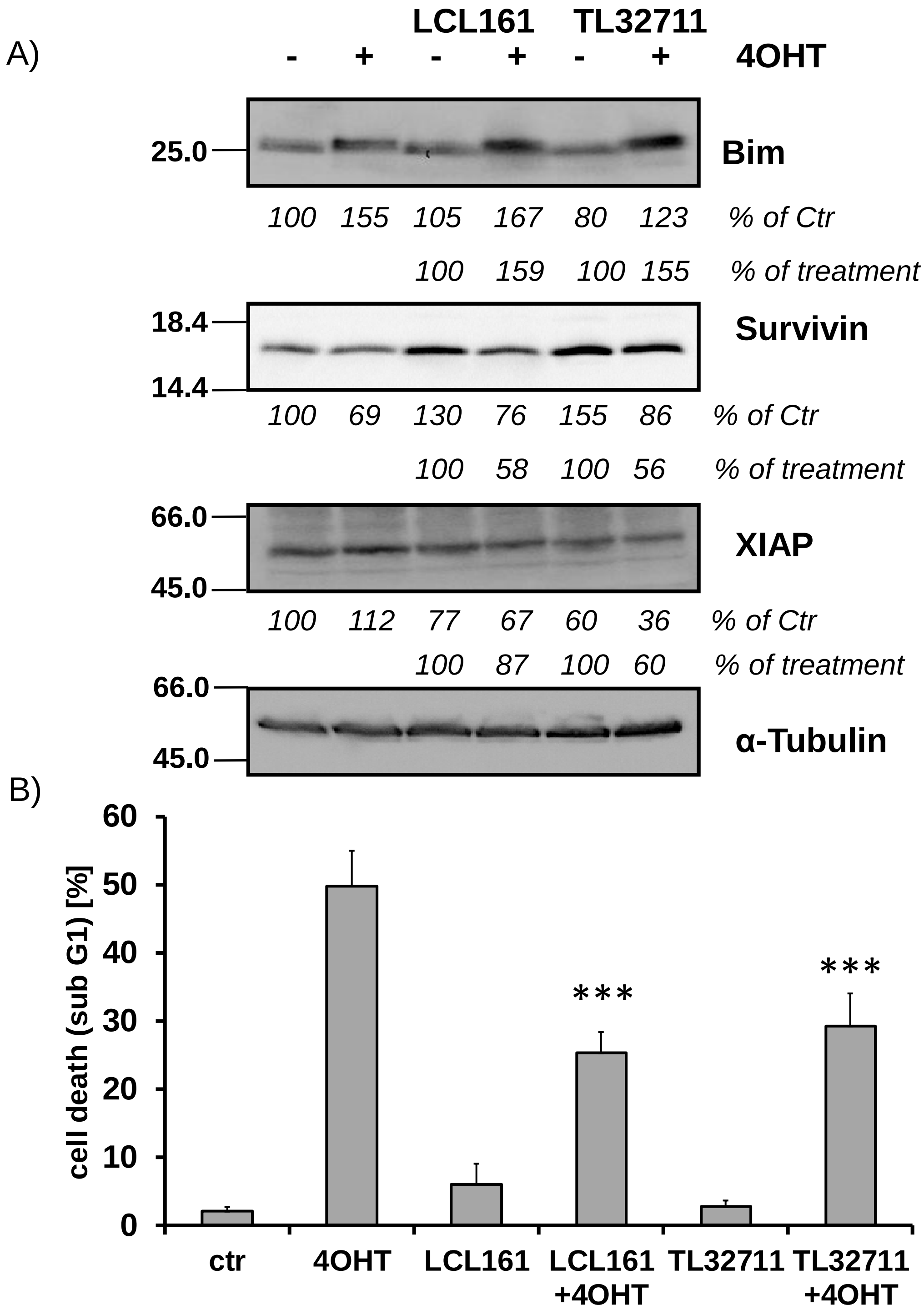

Figure S4: (A) Immunoblot analyses of SH-EP/FOXO3 cells treated with 4OHT for 8 hours alone or in combination with 10 µM LCL161 or TL32711 (pre-incubated for 24 hours). Cell lysates were subjected to immunoblot analyses against BIM, survivin, and XIAP. Tubulin served as loading control. (B) PI-FACS analyses of SH-EP/FOXO3 cells treated with 20 nM 4OHT for 24 hours alone or in combination with 10 µM LCL161 or TL32711, respectively (pre-incubated for 12 hours). Shown is the mean of five independent experiments. Statistical differences were assessed by unpaired t-test ***P<0.001.

Figure S5

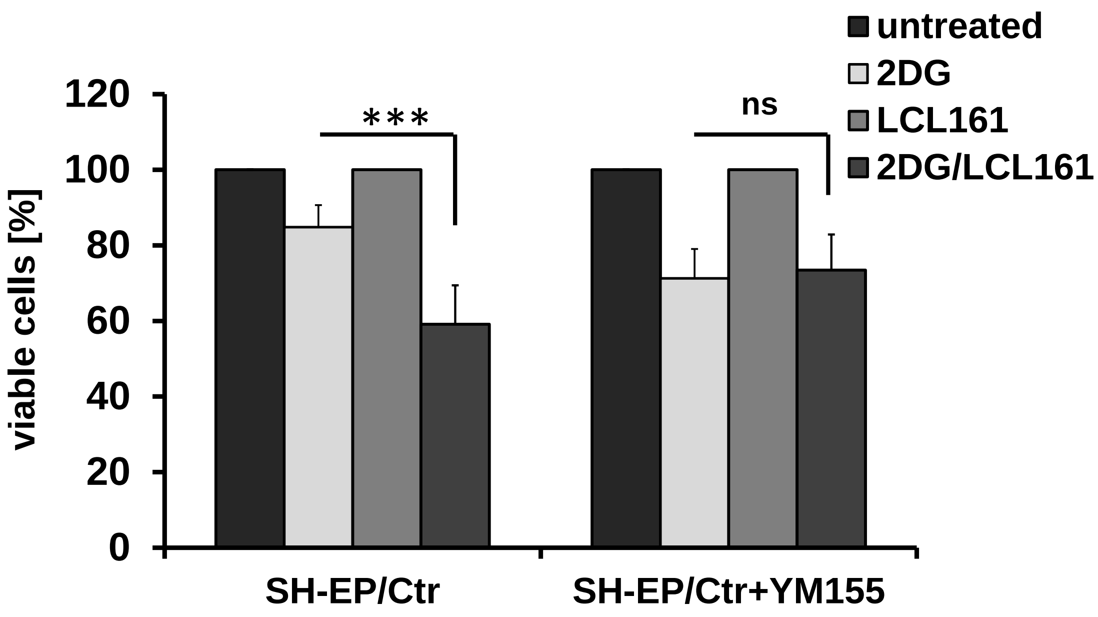

Figure S5: Viability analysis in SH-EP/Ctr cells treated with 5 mM 2DG for 24 hours. Cell were pre-incubated with 10 µM LCL161 alone or in combination with 5 nM YM155 for 12 hours. Shown is the mean+SD of three independent experiments. Statistical differences were assessed by unpaired t-test (***P<0.001).

Figure S6

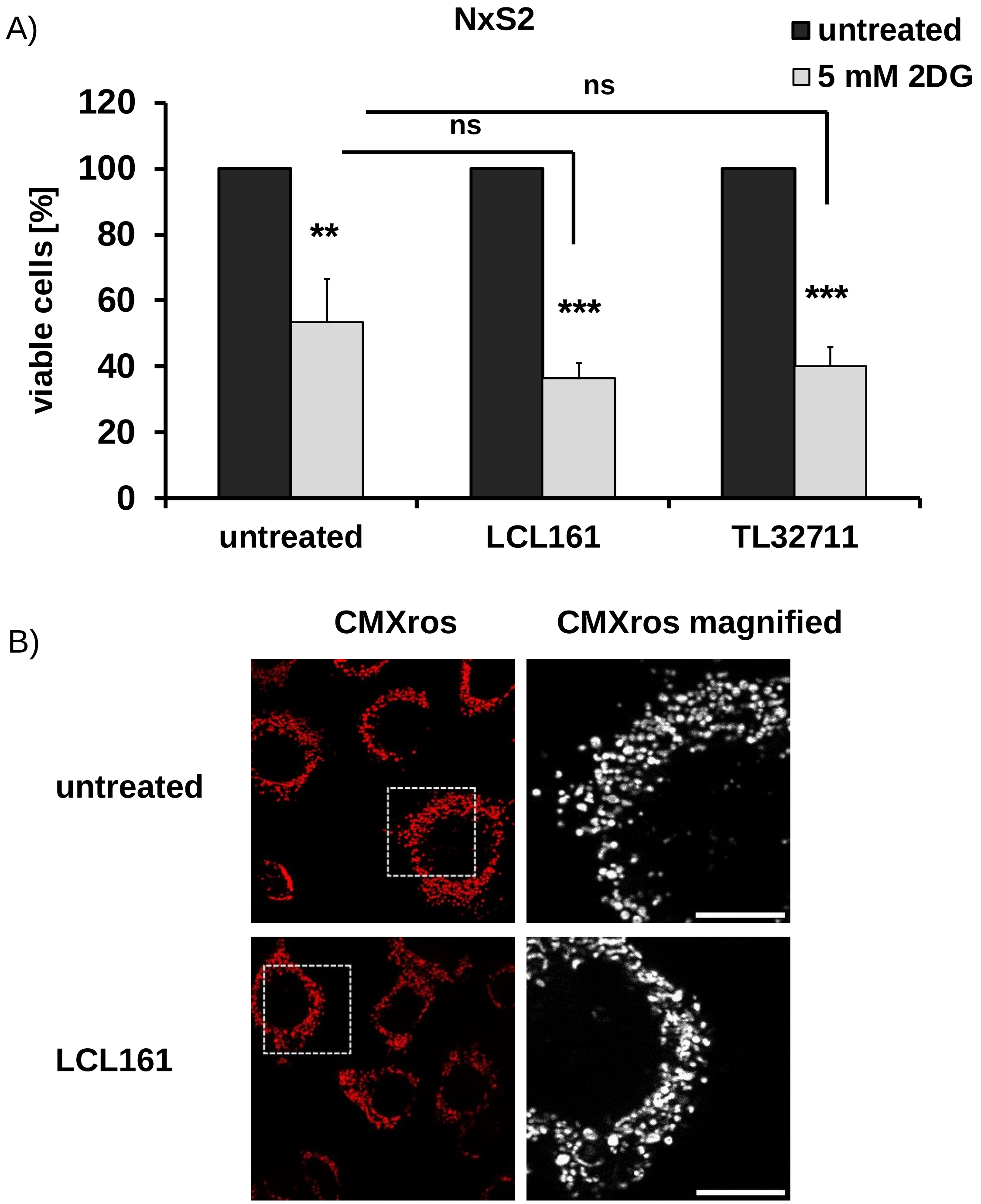

Figure S6: (A) Viability analyses after 24 hours of NxS2 cells after treatment with 5 mM 2DG alone or in combination with 20 µM LCL161 or TL32711 (pre-incubated for 12 hours). Shown is the mean of 3 independent experiments. **P<0.01; ***P<0.001. (B) Live cell imaging of NxS2 cells after mitochondrial staining with CMXRos (300 nM). Images were acquired with an Axiovert 200M Zeiss microscope equipped with an Apotome2.0, Bar is 10 µm.

Figure S6

C)

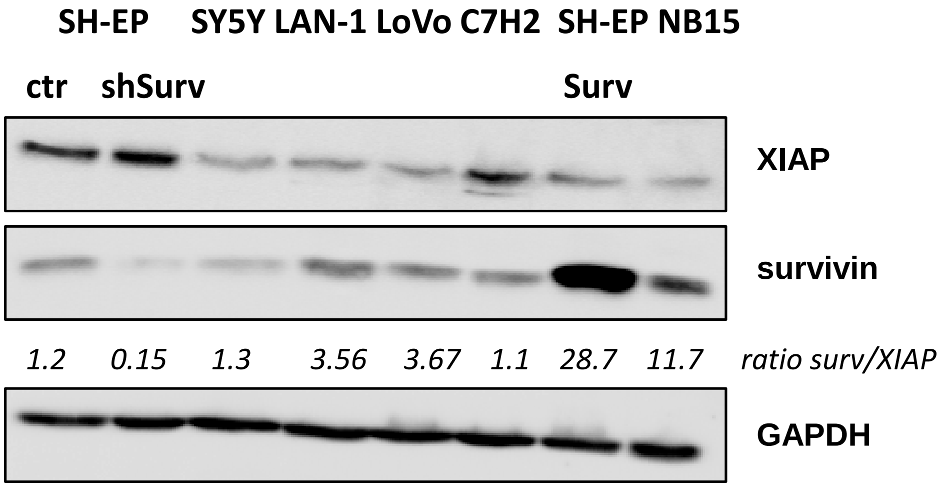

Figure S6: (C) Immunoblot analyses of the cells lines indicated. Immunoblots were incubated with antibodies against XIAP and survivin. GAPDH served as loading control. After normalization to GAPDH, the densitometric results were calculated as ratio between survivin and XIAP. The 17q amplified neuroblastoma cell line NB15 served as positive control.
